# Hydrogen-bonded mol­ecular salts of reduced benzo­thia­zole derivatives with carboxyl­ates: a robust 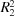
(8) supra­molecular motif (even when disordered)

**DOI:** 10.1107/S2056989018018224

**Published:** 2019-01-08

**Authors:** Mohammed A. E. Shaibah, Belakavadi K. Sagar, Hemmige S. Yathirajan, David B. Cordes, Alexandra M. Z. Slawin, William T. A. Harrison

**Affiliations:** a Department of Studies in Chemistry, University of Mysore, Manasagangotri, Mysuru 570 006, India; b School of Chemistry, University of St Andrews, Fife KY16 9ST, Scotland; c Department of Chemistry, University of Aberdeen, Meston Walk, Aberdeen AB24 3UE, Scotland

**Keywords:** benzo­thia­zole, hydrogen bond, mol­ecular salt, crystal structure

## Abstract

The syntheses and structures of five mol­ecular salts of protonated 4,4,7,7-tetra­methyl-3a,5,6,7a-tetra­hydro­benzo­thia­zol-2-yl­amine (C_11_H_19_N_2_S^+^) with different deprotonated carb­oxy­lic acids (4-methyl­benzoic acid, 4-bromo­benzoic acid, 3,5-di­nitro­benzoic acid, fumaric acid and succinic acid) are reported, namely 2-amino-4,4,7,7-tetra­methyl-4,5,6,7-tetra­hydro-1,3-benzo­thia­zol-3-ium 4-methyl­benzoate, C_11_H_19_N_2_S^+^·C_8_H_7_O_2_^−^, (I), 2-amino-4,4,7,7-tetra­methyl-4,5,6,7-tetra­hydro-1,3-benzo­thia­zol-3-ium 4-bromo­benzoate, C_11_H_19_N_2_S^+^·C_7_H_4_BrO_2_^−^, (II), 2-amino-4,4,7,7-tetra­methyl-4,5,6,7-tetra­hydro-1,3-benzo­thia­zol-3-ium 3,5-di­nitro­benzoate, C_11_H_19_N_2_S^+^·C_7_H_3_N_2_O_6_^−^, (III), bis­(2-amino-4,4,7,7-tetra­methyl-4,5,6,7-tetra­hydro-1,3-benzo­thia­zol-3-ium) fumarate, 2C_11_H_19_N_2_S^+^·C_4_H_2_O_4_^2−^,(IV), and the 1:1 co-crystal of bis­(2-amino-4,4,7,7-tetra­methyl-4,5,6,7-tetra­hydro-1,3-benzo­thia­zol-3-ium) succinate and 2-amino-4,4,7,7-tetra­methyl-4,5,6,7-tetra­hydro-1,3-benzo­thia­zol-3-ium hydrogen succin­ate 4,4,7,7-tetra­methyl-3a,5,6,7a-tetra­hydro­benzo­thia­zol-2-yl­amine, 1.5C_11_H_19_N_2_S^+^·0.5C_4_H_4_O_4_^2−^·0.5C_4_H_5_O_4_^−^. 0.5C_11_H_18_N_2_S, (V). In every case, the cation protonation occurs at the N atom of the thia­zole ring and the six-membered ring adopts a half-chair conformation (in some cases, the deviating methyl­ene groups are disordered over two sets of sites). The C—N bond lengths of the nominal –NH^+^=C—NH_2_ fragment of the cation are indistinguishable, indicating a significant contribution of the –NH—C=N^+^H_2_ resonance form to the structure. The packing for (I)–(V) features a robust local *R*_2_^2^(8) loop motif in which the cation forms two near-linear N—H⋯O hydrogen bonds from the N^+^—H group and *syn* H atom of the amine group to the carboxyl­ate group of an adjacent anion [(V) shows disorder of one of these bonds over N—H⋯O and N⋯H—O contributors but the same *R*_2_^2^(8) loop results for both disorder components]. The *anti* H atom of the –NH_2_ group also forms an N—H⋯O hydrogen bond, which results in [001] chains in (I) and (II), isolated centrosymmetric tetra­mers in (III) and [100] chains in (IV) and (V). Hirshfeld fingerprint plots and contact percentages for the different types of contacts of the cations are discussed.

## Abstract 2

The syntheses and structures of five mol­ecular salts of protonated 4,4,7,7-tetra­methyl-3a,5,6,7a-tetra­hydro­benzo­thia­zol-2-yl­amine (C_11_H_19_N_2_S^+^) with different deprotonated carb­oxy­lic acids (4-methyl­benzoic acid, 4-bromo­benzoic acid, 3,5-di­nitro­benzoic acid, fumaric acid and succinic acid) are reported·In every case, the cation protonation occurs at the N atom of the thia­zole ring and the six-membered ring adopts a half-chair conformation (in some cases, the deviating methyl­ene groups are disordered over two sets of sites). The C—N bond lengths of the nominal –NH^+^=C—NH_2_ fragment of the cation are indistinguishable, indicating a significant contribution of the –NH—C=N^+^H_2_ resonance form to the structure.

## Chemical context   

Some 2-amino­benzo­thia­zole derivatives display important biological properties: riluzole [2-amino-6-(tri­fluoro­meth­oxy)benzo­thia­zole] is used in the palliative treatment of amyotrophic lateral sclerosis (Sweeney *et al.*, 2018 ▸) and pramipexole di­hydro­chloride [(*S*)-*N*6-propyl-4,5,6,7-tetra­hydro­benzo[*d*]thia­zole-2,6-di­amine di­hydro­chloride] is used to combat Parkinson’s disease (Roy *et al.*, 2018 ▸). We note that the six-membered ring in the latter compound is reduced by the addition of four H atoms. In coordination chemistry, 2-amino­benzo­thia­zole has been shown to ligate to various metal ions, for example copper(II) (Kuwar *et al.*, 2018 ▸), cadmium(II) (Ma *et al.*, 2012 ▸) and palladium(II) (Gao *et al.*, 2011 ▸). The utility of 2-amino­benzo­thia­zole in organic synthesis has recently been reviewed (Dadmal *et al.*, 2018 ▸).
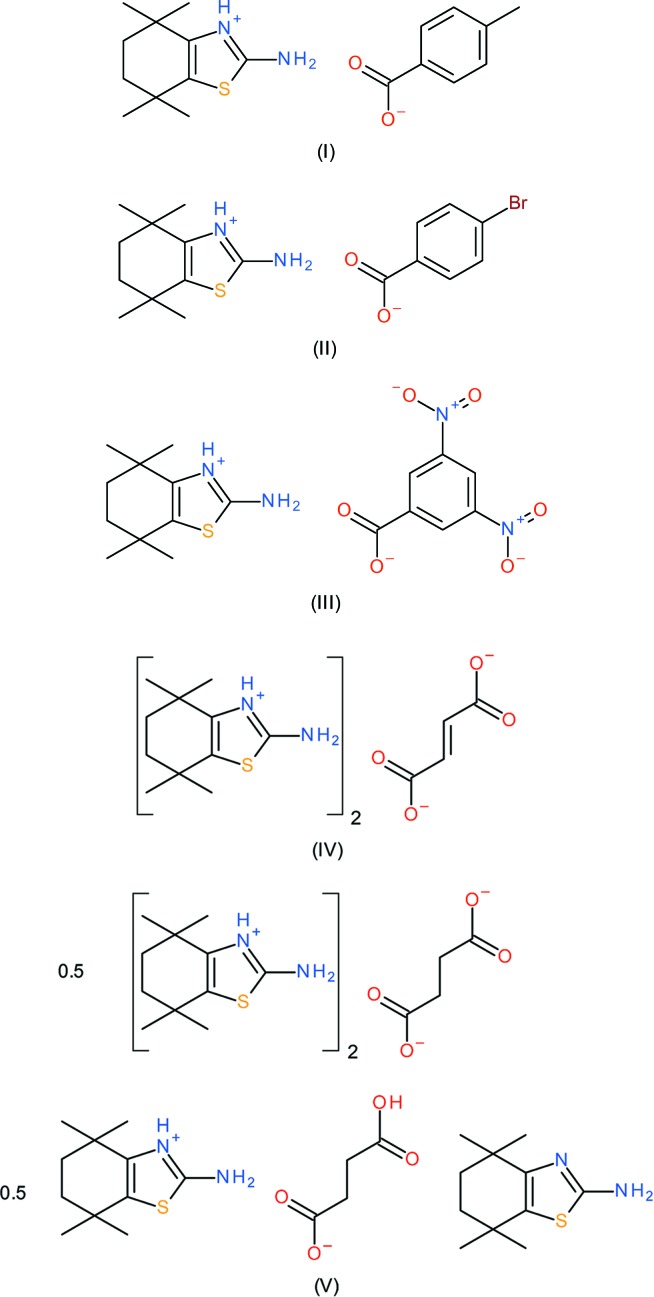


As part of our ongoing studies in this area (Sagar *et al.*, 2017 ▸), we now describe the syntheses and crystal structures of five mol­ecular salts of 4,4,7,7-tetra­methyl-3a,4,5,6,7,7a-hexa­hydro­benzo­thia­zol-2-yl­amine (C_11_H_18_N_2_S) with different carb­oxy­lic acids, viz. 2-amino-4,4,7,7-tetra­methyl-4,5,6,7-tetra­hydro-1,3-benzo­thia­zol-3-ium 4-methyl­benzoate, C_11_H_19_N_2_S^+^·C_8_H_7_O_2_^−^, (I); 2-amino-4,4,7,7-tetra­methyl-4,5,6,7-tetra­hydro-1,3-benzo­thia­zol-3-ium 4-bromo­benzoate, C_11_H_19_N_2_S^+^·C_7_H_4_BrO_2_^−^, (II); 2-amino-4,4,7,7-tetra­methyl-4,5,6,7-tetra­hydro-1,3-benzo­thia­zol-3-ium 3,5-di­nitro­benzoate, C_11_H_19_N_2_S^+^·C_7_H_3_N_2_O_6_^−^, (III); bis­(2-amino-4,4,7,7-tetra­methyl-4,5,6,7-tetra­hydro-1,3-benzo­thia­zol-3-ium) fumarate, (C_11_H_19_N_2_S^+^)_2_·C_4_H_2_O_4_^2−^,(IV); 1:1 co-crystal of bis­(2-amino-4,4,7,7-tetra­methyl-4,5,6,7-tetra­hydro-1,3-benzo­thia­zol-3-ium) succinate and (2-amino-4,4,7,7-tetra­methyl-4,5,6,7-tetra­hydro-1,3-benzo­thia­zol-3-ium) hydrogen succinate 4,4,7,7-tetra­methyl-3a,5,6,7a-tetra­hydro­benzo­thia­zol-2-yl­amine, (C_11_H_19_N_2_S^+^)_1.5_·(C_4_H_4_O_4_^2−^)_0.5_·(C_4_H_5_O_4_^−^)_0.5_·(C_11_H_18_N_2_S)_0.5_, (V).

## Structural commentary   

**Figure 1:**
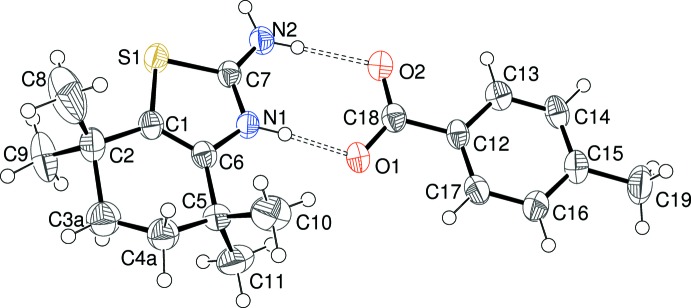
The mol­ecular structure of (I) showing the major disorder component only of the cation (50% displacement ellipsoids) with the hydrogen bonds indicated by double-dashed lines.

The asymmetric units of (I), (II) and (III) are illustrated in Figs. 1 ▸, 2 ▸ and 3 ▸, respectively. Each one features a 2-amino-4,4,7,7-tetra­methyl-4,5,6,7-tetra­hydro-1,3-benzo­thia­zol-3-ium (C_11_H_19_N_2_S^+^) cation protonated at the thia­zole-ring nitro­gen atom N1 accompanied by a substituted benzoate anion. The C7—N1 and C7—N2 bond lengths in the cations in (I) [1.325 (3) and 1.322 (4) Å, respectively], (II) [1.327 (5) and 1.316 (5) Å, respectively] and (III) [1.3267 (19) and 1.322 (2) Å, respectively] are almost identical, presumably indicating a significant contribution of the amidinium cation [—N1H—C7=N2^+^H_2_] resonance form to the overall structure [compare ions *A* and *B* in the chemical scheme of Sagar *et al.* (2017 ▸)]. The C1—S1—C7 bond angles [(I) = 90.27 (13); (II) = 90.29 (18); (III) = 90.40 (7)°] are almost identical in the three salts. The conformation of the reduced (hydrogenated/methyl­ated) six-membered ring of the benzo­thia­zole moiety can be described as a half-chair in each case; in (I), the deviating methyl­ene groups (C3 and C4) are disordered over two sets of sites in a 0.602 (10):0.398 (10) ratio. For (II), atoms C3 and C4 deviate from the plane defined by C1/C2/C5/C6 by −0.388 (13) and 0.130 (11) Å, respectively; comparable data for (III) are −0.276 (3) and 0.432 (3) Å, respectively. The dihedral angles between the C1/C2/C5/C6 moiety and the C1/C6/C7/N1/S1 thia­zole ring for (I) [1.3 (2)°], (II) [1.2 (3)°] and (III) [2.84 (13)°] indicate a slight, but statistically significant, puckering in each case.

In the substituted benzoate anion in (I), the C18—O1 [1.252 (3) Å] and C18—O2 [1.267 (3) Å] distances indicate substantial electronic delocalization within the carboxyl­ate group; the dihedral angle between the C12–C17 benzene ring and C18/O1/O2 is 7.0 (6)°. The equivalent data for (II) are C18—O1 = 1.260 (5), C18—O2 = 1.263 (5) Å and C12–C17 + C18/O1/O2 dihedral angle = 6.2 (8)°. The data for (III) are C18—O1 = 1.2475 (19), C18—O2 = 1.2506 (19) Å and dihedral angle = 4.9 (4)°. Additionally, the dihedral angles between the benzene ring and the N3 and N4 nitro groups in (III) are 16.0 (3) and 11.9 (3)°, respectively.

**Figure 4:**
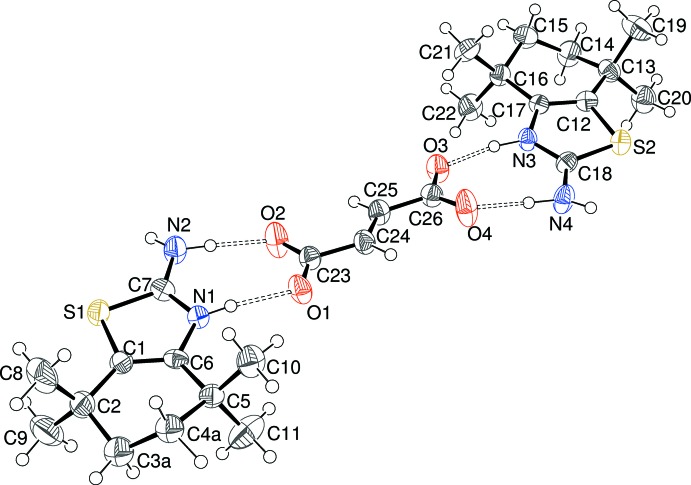
The mol­ecular structure of (IV) showing the major disorder component only (50% displacement ellipsoids) with the hydrogen bonds indicated by double-dashed lines.

The asymmetric unit of (IV) (Fig. 4 ▸) features two C_11_H_19_N_2_S^+^ cations and one dianion, which of course ensures charge balance. The structural features of the cations in (IV) are very similar to those of the equivalent species in (I)–(III): the C7—N1 and C7—N2 bond lengths of the S1 cation are 1.325 (6) and 1.318 (6) Å, respectively and the C18—N3 and C18—N4 bond lengths of the S2 cation are 1.320 (6) and 1.319 (7) Å, respectively and the same conclusion regarding delocal­ization of the [—N^+^1H=C7—N2H_2_] moiety as was noted for (I)–(III) can be drawn. The conformations of the six-membered rings are half-chairs in each case; in the N1 cation, the deviating methyl­ene groups (C3 and C4) are disordered over two sets of sites in a 0.78 (2):0.22 (2) ratio but these species are ordered in the N3 cation [deviations of C14 and C15 from the C12/C13/C16/C17 plane = 0.408 (11) and −0.309 (11) Å, respectively]. The fused rings are slightly puckered [dihedral angles (as defined above) = 0.7 (5) and 6.0 (3)° for the N1 and N3 cations, respectively]. In the fumarate anion in (IV), the carboxyl­ate bond lengths are C23—O1 = 1.251 (6), C23—O2 = 1.258 (6), C26—O3 = 1.267 (6) and C26—O4 = 1.232 (6) Å.

**Figure 5:**
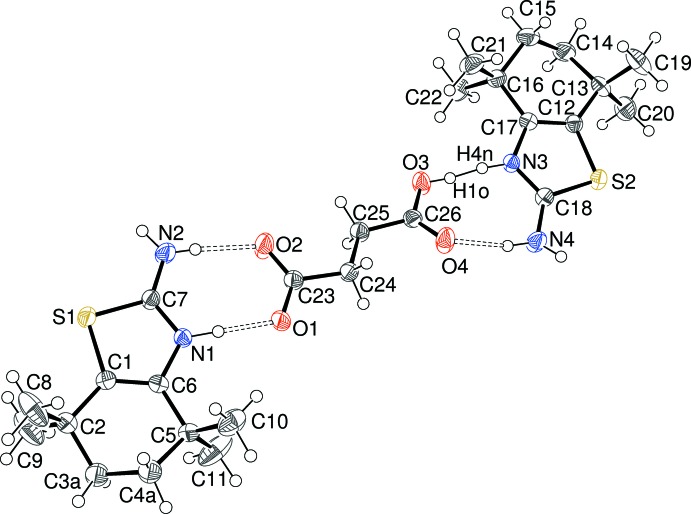
The mol­ecular structure of (V) showing the major disorder component of the methyl­ene groups only (50% displacement ellipsoids) and both disorder components for the N3—H⋯O3 and N3⋯H—O3 hydrogen bonds; the hydrogen bonds are indicated by double-dashed lines.

The asymmetric unit of (V) (Fig. 5 ▸) is more complex due to disorder of one of the transferable protons and can be envis­aged as a 1:1 co-crystal of (C_11_H_19_N_2_S^+^)_2_·(C_4_H_4_O_4_^2−^) *(i.e.* proton transfer from both carboxyl­ate groups of the anion) and (C_11_H_19_N_2_S^+^)·(C_4_H_5_O_4_^−^)(C_11_H_18_N_2_S) (*i.e.*: proton transfer from one of carboxyl­ate groups of the anion). This is further discussed below under supra­molecular features. Despite this disorder, the situation for the N1 and N3 cations in (V) is very similar to that in (IV): C7—N1 = 1.325 (3); C7—N2 = 1.317 (4); C18—N3 = 1.322 (3); C18—N4 = 1.336 (4) Å. The C1–C6 ring is disordered over two half-chair conformations in a 0.596 (11):0.404 (11) ratio but the C12–C17 ring is ordered with C14 and C15 deviating from the other atoms by 0.424 (6) and −0.313 (6) Å, respectively. The inter-ring dihedral angles are 0.6 (3) (N1 cation) and 1.3 (3)° (N3 cation). Key bond-length data for the succinate anion in (V) are C23—O1 = 1.254 (4), C23—O2 = 1.249 (4), C26—O3 = 1.284 (4) and C26—O4 = 1.229 (4) Å. These data indicate that the C—O single and double bonds within the C26/O3/O4 moiety are more localized than in the other structures reported here, which correlates with the proton disorder model associated with O3.

The ‘anomalous’ situation of incomplete proton transfer for (V) might be correlated with p*K*_a_ values for the acids involved: 4-methyl­benzoic acid (p*K*_a_ = 4.25), 4-bromo­benzoic acid (3.99), 3,5-di­nitro­benzoic acid (2.77), fumaric acid (p*K*_a1_ = 3.03, p*K*_a2_ = 4.54) and succinic acid (4.21, 5.64). These data apply to the acids dissolved in water (Jover *et al.*, 2008 ▸), but we might guess that a similar trend applies in the crystals and the p*K*_a2_ value for succinic acid is clearly larger than the others. Incomplete (or partial) proton transfer processes have been observed in other crystals (*e.g.* Biliškov *et al.*, 2011 ▸) and can lead to inter­esting physical properties (*e.g.* Noohinejad *et al.*, 2015 ▸).

## Supra­molecular features   

The most notable supra­molecular feature (which occur within the asymmetric units as defined here) of (I)–(V) is an 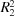
(8) loop in which the protonated N1^+^—H1 moiety of the thia­zole ring and the *syn* H atom of the –N2H_2_ amine group both form near-linear N—H⋯O hydrogen bonds to the O atoms of the carboxyl­ate group of an adjacent anion [Tables 1 ▸–5 ▸ ▸ ▸ ▸ for compounds (I)–(V), respectively]. In (V), the proton disorder associated with N3 and O3 leads to the same motif for both disorder components (two N—H⋯O bonds or one N—H⋯O and one N⋯H—O bond). Despite the presumed electronic delocalization of the cation noted above, it may be seen that for (I)–(III), the H⋯O distance for the charge-assisted hydrogen bond arising from N1 is notably shorter than the bond arising from N2. The situation for (IV) and (V) is less clear-cut: the H⋯O separations for the N1 and N2 (and equivalent N3 and N4) hydrogen bonds tend to be closer in magnitude and indeed the N2 bond in (IV) is marginally shorter than the N1 bond. The inter­molecular dihedral angles between the thia­zole and benzoate rings are 17.13 (14), 16.42 (19) and 20.15 (8)° for (I), (II) and (III), respectively, suggesting that the pairwise hydrogen bonds tend to align the aromatic rings of the cation and the anion in roughly the same plane.

**Figure 6:**
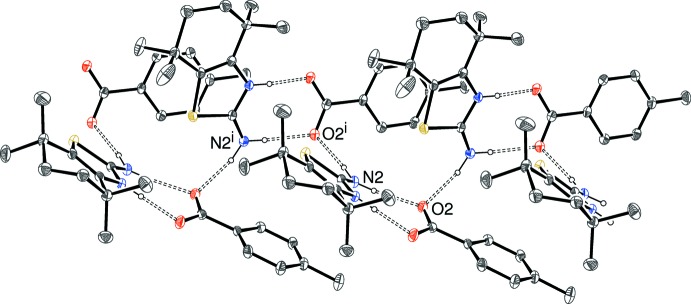
Fragment of an [001] hydrogen-bonded chain in the crystal of (I); the chain in (II) is almost identical to this. C-bound H atoms omitted for clarity. Symmetry code: (i) *x*, 1 − y, 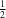 + *z*.

In every case, the amine N2–H3*N* group *anti* to the N^+^H group of the thia­zole ring also forms an N—H⋯O hydrogen bond, but the different anions lead to different overall structures. Salts (I) and (II) are isostructural (*i.e*. the same space group and packing with slight differences in the unit-cell parameters to accommodate the different para-substituents of the benzoate anion), with the N2—H3*N* group linking the ion pairs into [001] chains (Fig. 6 ▸), with adjacent mol­ecules related by *c*-glide symmetry. It may be noted that O2 accepts both hydrogen bonds from the amide H atoms and O1 accepts the charge-assisted bond from the thia­zole ring.

**Figure 7:**
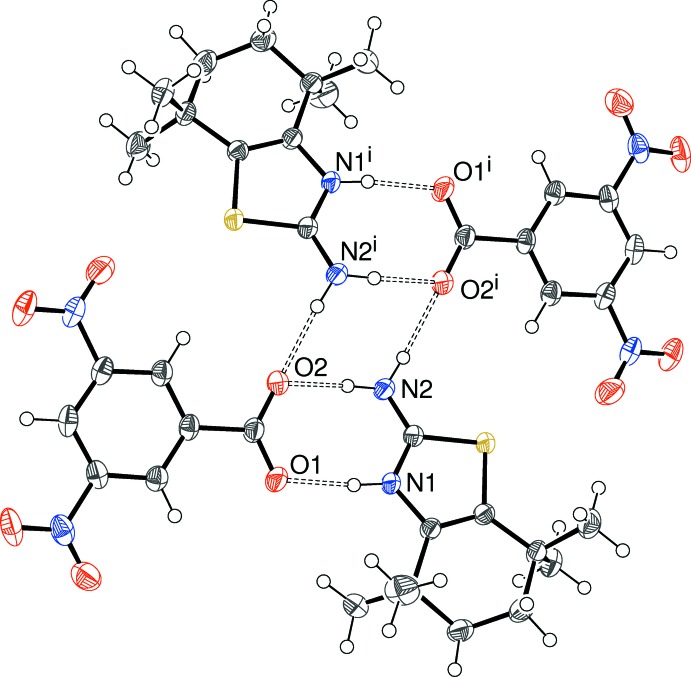
A centrosymmetric hydrogen-bonded tetra­mer in (III). Symmetry code: (i) 1 − x, 2 − y, 1 − z.

The situation for (III) is quite different, with isolated centrosymmetric tetra­mers (two cations and two anions) arising (Fig. 7 ▸) in which pairs of 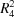
(8) loops linking one cation to two anions are apparent as well as the cation-to-anion 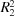
(8) loops already mentioned. A weak C—H⋯O inter­action (Table 3 ▸) arising from a methyl group occurs between tetra­mers.

**Figure 8:**
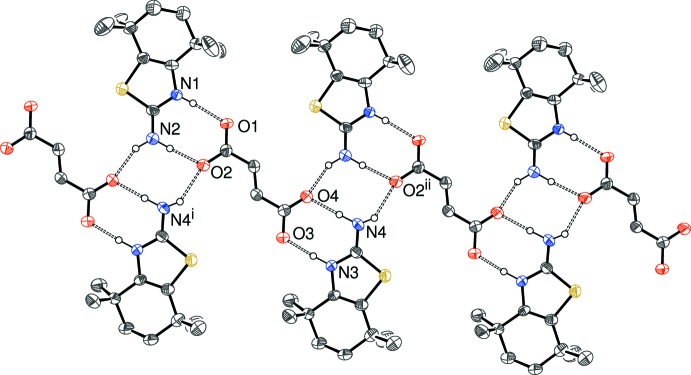
Fragment of a [100] hydrogen-bonded chain in the crystal of (IV); the chain in (V) is almost identical to this. C-bound H atoms omitted for clarity. Symmetry codes: (i) *x* + 1, *y*, *z*; (ii) *x* − 1, *y*, *z*.

Crystals (IV) and (V) are isostructural and feature [100] chains in each case (Fig. 8 ▸). It may be seen that locally the cation has the same hydrogen-bonding pattern to the anion as in (I) and (II) but because the dianions accept hydrogen bonds at ‘both ends’, a different overall structure arises, which features the same 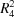
(8) loop seen in (III), but is not generated by a crystallographic centre of symmetry.

## Hirshfeld surface analyses   

**Figure 9:**
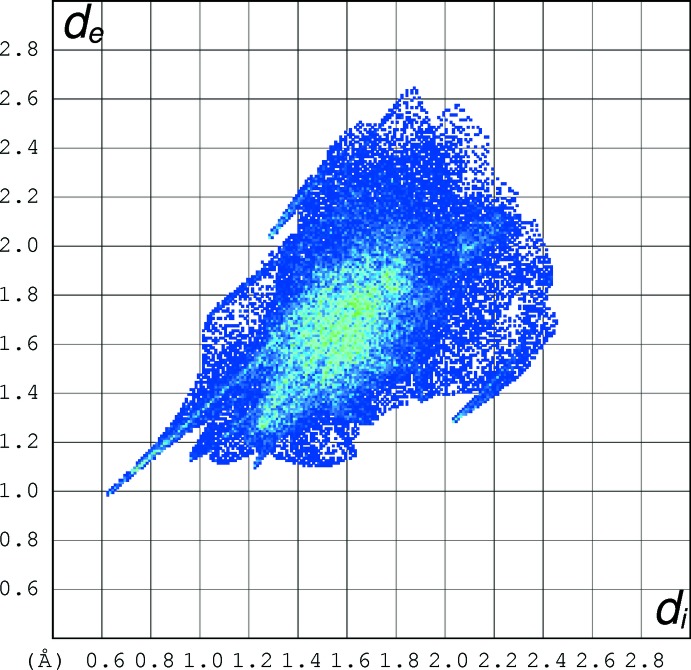
Hirshfeld fingerprint plot for (I).

The Hirshfeld surfaces of the C_11_H_19_N_2_S^+^ cations in (I)–(V) were calculated using *CrystalExplorer* (Turner *et al.*, 2017 ▸) and fingerprint plots (McKinnon *et al.*, 2007 ▸) were also generated. An example fingerprint plot for (I) is shown in Fig. 9 ▸; plots for (II)–(V) are available in the supporting information. The prominent ‘spike’ feature terminating at (*d*_i_, *d*_e_ = ∼0.62, 0.98) corresponds to the N—H⋯O hydrogen bonds. Less prominent spikes at (1.30, 2.05) and (2.05, 1.30) correspond to H⋯S and S⋯H contacts, respectively: if these are indicative of attractive directional inter­actions, they must be very weak at best, as the shortest H⋯S/S⋯H contact is 3.31 Å, compared to the van der Waals separation of 3.0 Å for these atoms.

The percentage surface contact data (Table 6 ▸) for the C_11_H_19_N_2_S^+^ species in the five structures reveal a number of similarities but also some differences: H⋯H contacts domin­ate the packing in each case, although the percentage for (III) is significantly less that for the others. The H⋯O contacts associated with the hydrogen bonds are very consistent for (I), (II), (IV) and (V), but those for (II) are much higher and presumably reflect the presence of the ‘extra’ O atoms of the nitro substituents of the anion, although no significant directional inter­actions could be identified for these O atoms apart from one weak C—H⋯O bond. The C⋯all, N⋯all and S⋯all contact percentages are almost identical for the five structures.

## Database survey   

So far as we are aware, the only reported crystal structures to contain the 4,4,7,7-tetra­methyl-3a,4,5,6,7,7a-hexa­hydro­benzo­thia­zol-2-yl­amine cation are those described recently by Sagar *et al.* (2017 ▸) (refcodes NEFTIE and NEFTOK), where it was crystallized with benzoate and picrate anions, respectively. The benzoate structure contains essentially the same hydrogen-bonded chains of cations and anions generated by *c*-glide symmetry as in (I) and (II) although it is not isostructural (space group *Cc* rather than *Pc*). The centrosymmetric, hydrogen-bonded tetra­mers in the picrate structure bear a resemblance to those in (III) but in the picrate anion, the acceptor oxygen atoms are the deprotonated phenol –OH group and adjacent nitro-group O atoms rather than carboxyl­ate O atoms.

A search of the Cambridge Structural Database (Groom *et al.*, 2016 ▸, updated to August 2018) for 2-amino­benzo­thia­zole with any substituents (including protonation) revealed 189 matches, but this number dropped to just five for a hydrogenated/methyl­ated six-membered ring, viz. (−)-2,6-di­amino-4,5,6,7-tetra­hydro­benzo­thia­zole (l)-(+)-tartrate trihydrate (refcode FECZES; Schneider & Mierau, 1987 ▸); *rac*-4,5,5a,6,7,8-hexa­hydro-6-*n*-propyl­thia­zolo(4,5-*f*)quinolin-2-amine methanol solvate (SONZIE; Caprathe *et al.*, 1991 ▸); 2-amino-5,6-di­hydro-1,3-benzo­thia­zol-7(4*H*)-one (TESGUV; Zhu *et al.*, 2012 ▸), as well as NEFTIE and NEFTOK referred to in the previous paragraph.

## Synthesis and crystallization   

4,4,7,7-Tetra­methyl-3a,4,5,6,7,7a-tetra­hydro­benzo­thia­zol-2-yl­amine (200 mg. 0.94 mmol) and the equivalent amount of the respective acids, *i.e.* 4-methyl­benzoic acid (135 mg, 0.94 mmol) for (I), 4-bromo­benzoic acid (200 mg, 0.94 mmol) for (II), 3,5-di­nitro­benzoic acid (208 mg, 0.94 mmol) for (III), fumaric acid (112 mg, 0.94 mmol) for (IV) and succinic acid (115 mg, 0.94 mmol) for (V), were dissolved in hot methanol and stirred over a heating magnetic stirrer for few minutes. The solution was allowed to cool slowly at room temperature and the resulting solids were recovered by filtration and drying in air. These were recrystallized at room temperature using a 1:1 solvent mixture of DMF and DMSO: crystals of (I) (m.p. 441–443 K), (II) (m.p. 473 K), (III) (m.p. 453–456 K), (IV) (m.p. 446–450 K) and (V) (m.p. 393–396 K) appeared after a week.

## Refinement   

Crystal data, data collection and structure refinement details are summarized in Table 7 ▸. The methyl­ene groups in (I), (IV) and (V) were modelled as being disordered over two sets of sites and the C atoms were refined with isotropic displacement parameters. The N-bound H atoms were located in difference-Fourier maps and their positions were freely refined without difficulty in every case except for the proton associated with atoms N3 and O3 in compound (V). Careful scrutiny of difference maps indicated two electron density maxima in the vicinity of these two atoms, one in a reasonable location for an N3—H⋯O3 hydrogen bond (*i.e*. proton transferred) and the other corres­ponding to an N3⋯H—O3 hydrogen bond (*i.e.* proton not transferred): for a detailed discussion of proton location in potentially disordered hydrogen bonds, see Fábry (2018 ▸). Despite their feeble scattering power, when included in the atomic model these refined well as disordered H atoms: their occupancy sum was constrained to unity and revealed equal occupancies [0.50 (5):0.50 (5)] for the two sites. H atoms for all structures were placed geometrically (C—H = 0.95–0.99 Å) and refined as riding atoms. The methyl groups were allowed to rotate, but not to tip, to best fit the electron density. In every case, the constraint *U*_iso_(H) = 1.2*U*_eq_(carrier) or 1.5*U*_eq_(methyl carrier) was applied. The absolute structures of (I), (II), (IV) and (V) were established by refinement of the Flack absolute structure parameter (Parsons *et al.*, 2013 ▸). It may be noted that despite being isostrutural, the crystal of (IV) chosen for data collection was found to be an inversion twin, whereas the chosen crystal of (V) has a well-defined absolute structure (despite disorder).

## Supplementary Material

Crystal structure: contains datablock(s) I, II, III, IV, V, global. DOI: 10.1107/S2056989018018224/mw2140sup1.cif

Structure factors: contains datablock(s) I. DOI: 10.1107/S2056989018018224/mw2140Isup2.hkl

Structure factors: contains datablock(s) II. DOI: 10.1107/S2056989018018224/mw2140IIsup3.hkl

Structure factors: contains datablock(s) III. DOI: 10.1107/S2056989018018224/mw2140IIIsup4.hkl

Structure factors: contains datablock(s) IV. DOI: 10.1107/S2056989018018224/mw2140IVsup5.hkl

Structure factors: contains datablock(s) V. DOI: 10.1107/S2056989018018224/mw2140Vsup6.hkl

Supporting information file. DOI: 10.1107/S2056989018018224/mw2140Isup7.cml

Supporting information file. DOI: 10.1107/S2056989018018224/mw2140IIsup8.cml

Supporting information file. DOI: 10.1107/S2056989018018224/mw2140IIIsup9.cml

Supporting information file. DOI: 10.1107/S2056989018018224/mw2140IVsup10.cml

CCDC references: 1886885, 1886884, 1886883, 1886882, 1886881

Additional supporting information:  crystallographic information; 3D view; checkCIF report

## Figures and Tables

**Figure 2:**
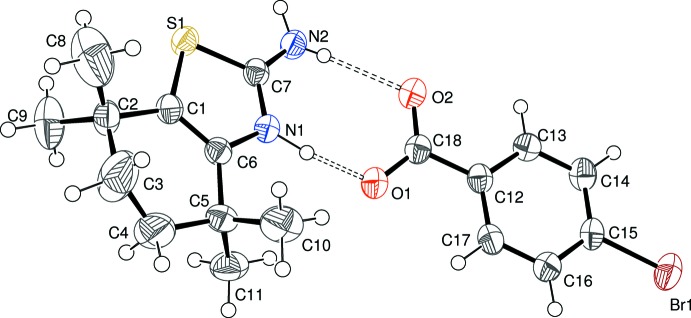
The mol­ecular structure of (II) showing 50% displacement ellipsoids with the hydrogen bonds indicated by double-dashed lines.

**Figure 3:**
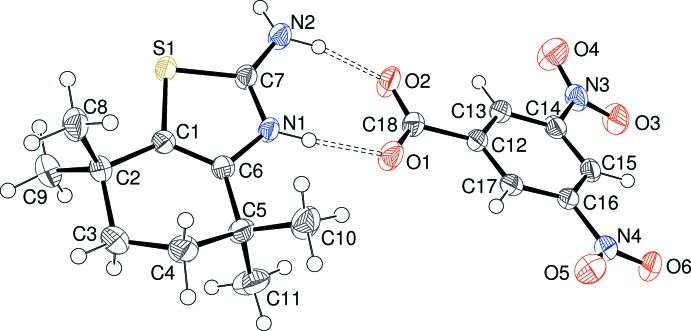
The mol­ecular structure of (III) showing 50% displacement ellipsoids with the hydrogen bonds indicated by double-dashed lines.

**Table 1:** Hydrogen-bond geometry (Å, °) for (I)

*D*—H⋯*A*	*D*—H	H⋯*A*	*D*⋯*A*	*D*—H⋯*A*
N1—H1*N*⋯O1	0.88 (3)	1.76 (3)	2.632 (3)	175 (3)
N2—H2*N*⋯O2	0.83 (3)	1.98 (4)	2.801 (3)	170 (3)
N2—H3*N*⋯O2^i^	0.89 (3)	1.93 (3)	2.805 (3)	164 (3)

Symmetry code: (i) 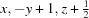.

**Table 2:** Hydrogen-bond geometry (Å, °) for (II)

*D*—H⋯*A*	*D*—H	H⋯*A*	*D*⋯*A*	*D*—H⋯*A*
N1—H1*N*⋯O1	1.01 (5)	1.62 (5)	2.626 (4)	172 (4)
N2—H2*N*⋯O2	0.72 (5)	2.12 (5)	2.816 (5)	162 (6)
N2—H3*N*⋯O2^i^	0.88 (5)	1.94 (5)	2.808 (5)	170 (4)

Symmetry code: (i) 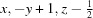.

**Table 3:** Hydrogen-bond geometry (Å, °) for (III)

*D*—H⋯*A*	*D*—H	H⋯*A*	*D*⋯*A*	*D*—H⋯*A*
N1—H1*N*⋯O1	0.885 (19)	1.838 (19)	2.7187 (17)	173.4 (17)
N2—H3*N*⋯O2^i^	0.90 (2)	2.01 (2)	2.8509 (19)	156.3 (17)
N2—H2*N*⋯O2	0.91 (2)	1.86 (2)	2.7551 (18)	168.3 (18)
C8—H8*A*⋯O6^ii^	0.98	2.43	3.397 (2)	170

Symmetry codes: (i) 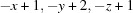; (ii) 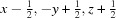.

**Table 4:** Hydrogen-bond geometry (Å, °) for (IV)

*D*—H⋯*A*	*D*—H	H⋯*A*	*D*⋯*A*	*D*—H⋯*A*
N1—H1*N*⋯O1	0.85 (5)	1.84 (5)	2.670 (5)	167 (5)
N2—H2*N*⋯O2	0.94 (6)	1.77 (6)	2.704 (6)	177 (5)
N2—H3*N*⋯O4^i^	0.91 (6)	1.90 (6)	2.746 (6)	153 (5)
N3—H4*N*⋯O3	0.96 (5)	1.67 (5)	2.617 (5)	169 (5)
N4—H5*N*⋯O4	1.00 (6)	1.75 (6)	2.754 (7)	178 (5)
N4—H6*N*⋯O2^ii^	0.81 (6)	2.09 (6)	2.763 (6)	141 (6)

Symmetry codes: (i) 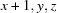; (ii) 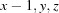.

**Table 5:** Hydrogen-bond geometry (Å, °) for (V)

*D*—H⋯*A*	*D*—H	H⋯*A*	*D*⋯*A*	*D*—H⋯*A*
N1—H1*N*⋯O1	0.93 (3)	1.70 (3)	2.632 (3)	175 (3)
N2—H2*N*⋯O4^i^	0.89 (4)	1.98 (4)	2.779 (3)	149 (3)
N2—H3*N*⋯O2	0.82 (4)	1.89 (4)	2.716 (4)	176 (4)
N3—H4*N*⋯O3	0.87 (8)	1.73 (8)	2.594 (3)	166 (6)
N4—H5*N*⋯O2^ii^	0.82 (4)	2.07 (4)	2.804 (4)	148 (4)
N4—H6*N*⋯O4	0.92 (4)	1.93 (4)	2.842 (4)	169 (3)
O3—H1*O*⋯N3	0.82 (10)	1.78 (10)	2.594 (3)	174 (8)

Symmetry codes: (i) 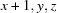; (ii) 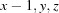.

**Table 6:** Hirshfeld contact inter­actions arising from the C_11_H_19_N_2_S^+^ cation (%) in (I)–(V)

Contact type	(I)	(II)	(III)	(IV)*a*	(IV)*b*	(V)*a*	(V)*b*
H⋯H	68.7	60.9	47.6	62.2	63.3	65.2	65.4
H⋯Br	–	6.2	–	–	–	–	–
H⋯O	10.9	12.0	29.1	13.5	13.0	13.4	12.0
H⋯C	5.9	6.7	6.6	6.0	5.5	3.7	4.2
H⋯S	2.1	1.8	1.6	3.5	3.0	3.3	2.6
C⋯all	2.8	2.9	2.9	3.1	3.1	3.1	3.0
N⋯all	2.7	2.7	2.7	3.0	2.9	2.8	3.2
S⋯all	6.5	6.3	6.4	6.2	6.4	6.2	6.5

‘*a*’ refers to the S1-containing cation and ‘*b*’ to the S2-cation.

**Table:** 

	(I)	(II)	(III)
Crystal data			
Chemical formula	C_11_H_19_N_2_S^+^·C_8_H_7_O_2_^−^	C_11_H_19_N_2_S^+^·C_7_H_4_BrO_2_^−^	C_11_H_19_N_2_S^+^·C_7_H_3_N_2_O_6_^−^
*M*_r_	346.48	411.35	422.45
Crystal system, space group	Monoclinic, *P* *c*	Monoclinic, *P* *c*	Monoclinic, *P*2_1_/*n*
Temperature (K)	173	173	173
*a*, *b*, *c* (Å)	10.1879 (5), 11.6149 (5), 8.7790 (4)	10.2506 (5), 11.5855 (4), 8.8269 (4)	17.3232 (6), 5.84087 (17), 21.0876 (8)
β (°)	113.480 (6)	113.807 (6)	109.875 (4)
*V* (Å^3^)	952.82 (9)	959.07 (8)	2006.60 (12)
*Z*	2	2	4
Radiation type	Mo *K*α	Mo *K*α	Mo *K*α
μ (mm^−1^)	0.18	2.26	0.21
Crystal size (mm)	0.24 × 0.11 × 0.02	0.12 × 0.08 × 0.02	0.27 × 0.10 × 0.03

Data collection			
Diffractometer	Rigaku XtaLAB P200 HPC	Rigaku XtaLAB P200 HPC	Rigaku XtaLAB P200 HPC
Absorption correction	Multi-scan (*CrysAlis PRO*; Rigaku, 2017 ▸)	Multi-scan (*CrysAlis PRO*; Rigaku, 2017 ▸)	Multi-scan (*CrysAlis PRO*; Rigaku, 2017 ▸)
*T*_min_, *T*_max_	0.793, 1.000	0.761, 1.000	0.566, 1.000
No. of measured, independent and observed [*I* > 2σ(*I*)] reflections	12189, 4056, 3440	12220, 3993, 3311	25034, 4698, 3712
*R*_int_	0.029	0.026	0.056
(sin θ/λ)_max_ (Å^−1^)	0.683	0.683	0.686

Refinement			
*R*[*F* ^2^ > 2σ(*F* ^2^)], *wR*(*F* ^2^), *S*	0.038, 0.089, 1.03	0.032, 0.083, 1.06	0.042, 0.116, 1.05
No. of reflections	4056	3993	4698
No. of parameters	230	230	275
No. of restraints	2	2	0
H-atom treatment	H atoms treated by a mixture of independent and constrained refinement	H atoms treated by a mixture of independent and constrained refinement	H atoms treated by a mixture of independent and constrained refinement
Δρ_max_, Δρ_min_ (e Å^−3^)	0.20, −0.17	0.62, −0.38	0.49, −0.25
Absolute structure	Flack *x* determined using 1371 quotients [(*I* ^+^)−(*I* ^−^)]/[(*I* ^+^)+(*I* ^−^)] (Parsons *et al.*, 2013 ▸).	Flack *x* determined using 1344 quotients [(*I* ^+^)−(*I* ^−^)]/[(*I* ^+^)+(*I* ^−^)] (Parsons *et al.*, 2013 ▸).	–
Absolute structure parameter	0.03 (3)	0.005 (4)	–

**Table:** 

	(IV)	(V)
Crystal data		
Chemical formula	2C_11_H_19_N_2_S^+^·C_4_H_2_O_4_^2−^	1.5C_11_H_19_N_2_S^+^·0.5C_4_H_4_O_4_^2−^·0.5C_4_H_5_O_4_^−^·0.5C_11_H_18_N_2_S
*M*_r_	536.74	538.75
Crystal system, space group	Monoclinic, *P*2_1_	Monoclinic, *P*2_1_
Temperature (K)	173	173
*a*, *b*, *c* (Å)	9.0259 (6), 14.7314 (11), 11.1993 (8)	8.9437 (3), 14.7253 (4), 11.2676 (4)
β (°)	101.943 (6)	100.493 (3)
*V* (Å^3^)	1456.87 (18)	1459.11 (8)
*Z*	2	2
Radiation type	Mo *K*α	Mo *K*α
μ (mm^−1^)	0.22	0.22
Crystal size (mm)	0.15 × 0.08 × 0.02	0.32 × 0.15 × 0.02

Data collection		
Diffractometer	Rigaku XtaLAB P200 HPC	Rigaku XtaLAB P200 HPC
Absorption correction	Multi-scan (*CrysAlis PRO*; Rigaku, 2017 ▸)	Multi-scan (*CrysAlis PRO*; Rigaku, 2017 ▸)
*T*_min_, *T*_max_	0.333, 1.000	0.779, 1.000
No. of measured, independent and observed [*I* > 2σ(*I*)] reflections	19263, 6424, 3792	19079, 6272, 5511
*R*_int_	0.080	0.034
(sin θ/λ)_max_ (Å^−1^)	0.684	0.685

Refinement		
*R*[*F* ^2^ > 2σ(*F* ^2^)], *wR*(*F* ^2^), *S*	0.062, 0.128, 1.00	0.039, 0.094, 1.04
No. of reflections	6424	6272
No. of parameters	350	354
No. of restraints	1	1
H-atom treatment	H atoms treated by a mixture of independent and constrained refinement	H atoms treated by a mixture of independent and constrained refinement
Δρ_max_, Δρ_min_ (e Å^−3^)	0.27, −0.26	0.26, −0.25
Absolute structure	Flack *x* determined using 1279 quotients [(*I* ^+^)−(*I* ^−^)]/[(*I* ^+^)+(*I* ^−^)] (Parsons *et al.*, 2013 ▸).	Flack *x* determined using 2211 quotients [(*I* ^+^)−(*I* ^−^)]/[(*I* ^+^)+(*I* ^−^)] (Parsons *et al.*, 2013 ▸)
Absolute structure parameter	0.46 (7)	0.00 (3)

Computer programs: *CrystalClear-SM* (Rigaku, 2017 ▸), *CrysAlis PRO* (Rigaku, 2017 ▸), *SHELXS97* (Sheldrick, 2008 ▸), *SHELXL2014* (Sheldrick, 2015 ▸), *ORTEP-3 for Windows* (Farrugia, 2012 ▸) and *publCIF* (Westrip, 2010 ▸).

## supplementary crystallographic information

### 2-Amino-4,4,7,7-tetramethyl-3a,5,6,7a-tetrahydrobenzothiazol-3-ium 4-methylbenzoate (I) Crystal data

**Table:**

C_11_H_19_N_2_S^+^·C_8_H_7_O_2_^−^	*F*(000) = 372
*M**_r_* = 346.48	*D*_x_ = 1.208 Mg m^−^^3^
Monoclinic, *P**c*	Mo *K*α radiation, λ = 0.71073 Å
*a* = 10.1879 (5) Å	Cell parameters from 5914 reflections
*b* = 11.6149 (5) Å	θ = 2.2–28.1°
*c* = 8.7790 (4) Å	µ = 0.18 mm^−^^1^
β = 113.480 (6)°	*T* = 173 K
*V* = 952.82 (9) Å^3^	Plate, colourless
*Z* = 2	0.24 × 0.11 × 0.02 mm

### 2-Amino-4,4,7,7-tetramethyl-3a,5,6,7a-tetrahydrobenzothiazol-3-ium 4-methylbenzoate (I) Data collection

**Table:**

Rigaku XtaLAB P200 HPC diffractometer	4056 independent reflections
Radiation source: rotating anode, Rigaku FR-X	3440 reflections with *I* > 2σ(*I*)
Rigaku Osmic Confocal Optical System monochromator	*R*_int_ = 0.029
ω scans	θ_max_ = 29.0°, θ_min_ = 1.8°
Absorption correction: multi-scan (CrysAlis PRO; Rigaku, 2017)	*h* = −13→12
*T*_min_ = 0.793, *T*_max_ = 1.000	*k* = −15→15
12189 measured reflections	*l* = −11→11

### 2-Amino-4,4,7,7-tetramethyl-3a,5,6,7a-tetrahydrobenzothiazol-3-ium 4-methylbenzoate (I) Refinement

**Table:**

Refinement on *F*^2^	Hydrogen site location: mixed
Least-squares matrix: full	H atoms treated by a mixture of independent and constrained refinement
*R*[*F*^2^ > 2σ(*F*^2^)] = 0.038	*w* = 1/[σ^2^(*F*_o_^2^) + (0.0463*P*)^2^ + 0.0807*P*] where *P* = (*F*_o_^2^ + 2*F*_c_^2^)/3
*wR*(*F*^2^) = 0.089	(Δ/σ)_max_ < 0.001
*S* = 1.03	Δρ_max_ = 0.20 e Å^−^^3^
4056 reflections	Δρ_min_ = −0.17 e Å^−^^3^
230 parameters	Absolute structure: Flack *x* determined using 1371 quotients [(*I*^+^)-(*I*^-^)]/[(*I*^+^)+(*I*^-^)] (Parsons *et al.*, 2013).
2 restraints	Absolute structure parameter: 0.03 (3)
Primary atom site location: structure-invariant direct methods	

### 2-Amino-4,4,7,7-tetramethyl-3a,5,6,7a-tetrahydrobenzothiazol-3-ium 4-methylbenzoate (I) Special details

**Table:**

Geometry. All esds (except the esd in the dihedral angle between two l.s. planes) are estimated using the full covariance matrix. The cell esds are taken into account individually in the estimation of esds in distances, angles and torsion angles; correlations between esds in cell parameters are only used when they are defined by crystal symmetry. An approximate (isotropic) treatment of cell esds is used for estimating esds involving l.s. planes.

### 2-Amino-4,4,7,7-tetramethyl-3a,5,6,7a-tetrahydrobenzothiazol-3-ium 4-methylbenzoate (I) Fractional atomic coordinates and isotropic or equivalent isotropic displacement parameters (Å^2^)

**Table:**

	*x*	*y*	*z*	*U*_iso_*/*U*_eq_	Occ. (<1)
C1	0.3617 (3)	0.6584 (2)	0.8458 (3)	0.0363 (6)	
C2	0.2322 (3)	0.6675 (3)	0.8865 (4)	0.0466 (7)	
C3A	0.1685 (10)	0.7939 (8)	0.8152 (12)	0.058 (3)*	0.398 (10)
H3A1	0.0740	0.8028	0.8210	0.069*	0.398 (10)
H3A2	0.2329	0.8534	0.8878	0.069*	0.398 (10)
C4A	0.1525 (10)	0.8143 (9)	0.6457 (13)	0.048 (2)*	0.398 (10)
H4A1	0.1003	0.8874	0.6050	0.058*	0.398 (10)
H4A2	0.0957	0.7512	0.5735	0.058*	0.398 (10)
C3B	0.1217 (6)	0.7333 (5)	0.7440 (7)	0.0467 (18)*	0.602 (10)
H3B1	0.0401	0.7535	0.7730	0.056*	0.602 (10)
H3B2	0.0852	0.6836	0.6441	0.056*	0.602 (10)
C4B	0.1860 (7)	0.8447 (6)	0.7047 (9)	0.0468 (15)*	0.602 (10)
H4B1	0.1081	0.8907	0.6225	0.056*	0.602 (10)
H4B2	0.2291	0.8911	0.8074	0.056*	0.602 (10)
C5	0.3013 (3)	0.8207 (2)	0.6356 (4)	0.0412 (7)	
C6	0.3917 (3)	0.7225 (2)	0.7371 (3)	0.0342 (6)	
C7	0.5894 (3)	0.6071 (2)	0.8256 (3)	0.0341 (6)	
C8	0.1541 (6)	0.5542 (4)	0.8622 (7)	0.111 (2)	
H8A	0.0673	0.5643	0.8833	0.167*	
H8B	0.2164	0.4971	0.9397	0.167*	
H8C	0.1280	0.5275	0.7480	0.167*	
C9	0.2747 (4)	0.7056 (4)	1.0652 (5)	0.0783 (12)	
H9A	0.1884	0.7226	1.0847	0.117*	
H9B	0.3342	0.7749	1.0862	0.117*	
H9C	0.3288	0.6440	1.1401	0.117*	
C10	0.2527 (4)	0.7945 (3)	0.4504 (4)	0.0648 (10)	
H10A	0.1905	0.8566	0.3853	0.097*	
H10B	0.2000	0.7216	0.4246	0.097*	
H10C	0.3366	0.7886	0.4227	0.097*	
C11	0.3888 (4)	0.9315 (3)	0.6748 (5)	0.0622 (9)	
H11A	0.4216	0.9487	0.7935	0.093*	
H11B	0.3293	0.9950	0.6100	0.093*	
H11C	0.4718	0.9220	0.6461	0.093*	
N1	0.5199 (2)	0.69242 (19)	0.7257 (3)	0.0352 (5)	
H1N	0.559 (3)	0.719 (3)	0.660 (4)	0.042*	
N2	0.7129 (3)	0.5644 (2)	0.8356 (3)	0.0406 (6)	
H2N	0.738 (3)	0.583 (3)	0.759 (4)	0.049*	
H3N	0.742 (3)	0.502 (3)	0.900 (4)	0.049*	
S1	0.49793 (8)	0.55753 (6)	0.94144 (8)	0.04197 (19)	
C12	0.7414 (3)	0.7561 (2)	0.3383 (3)	0.0353 (6)	
C13	0.8440 (3)	0.6995 (3)	0.2992 (3)	0.0393 (6)	
H13	0.8915	0.6339	0.3613	0.047*	
C14	0.8774 (3)	0.7387 (3)	0.1694 (3)	0.0428 (7)	
H14	0.9470	0.6986	0.1432	0.051*	
C15	0.8121 (3)	0.8343 (3)	0.0779 (4)	0.0457 (7)	
C16	0.7089 (4)	0.8898 (3)	0.1168 (4)	0.0533 (8)	
H16	0.6618	0.9556	0.0547	0.064*	
C17	0.6734 (3)	0.8509 (3)	0.2443 (4)	0.0474 (8)	
H17	0.6015	0.8898	0.2678	0.057*	
C18	0.7053 (3)	0.7165 (2)	0.4803 (3)	0.0382 (6)	
C19	0.8510 (4)	0.8777 (4)	−0.0609 (4)	0.0688 (10)	
H19A	0.8263	0.9595	−0.0806	0.103*	
H19B	0.7981	0.8339	−0.1624	0.103*	
H19C	0.9541	0.8680	−0.0298	0.103*	
O1	0.6233 (2)	0.77900 (19)	0.5192 (3)	0.0518 (5)	
O2	0.7604 (2)	0.62322 (16)	0.5523 (2)	0.0428 (5)	

### 2-Amino-4,4,7,7-tetramethyl-3a,5,6,7a-tetrahydrobenzothiazol-3-ium 4-methylbenzoate (I) Atomic displacement parameters (Å^2^)

**Table:**

	*U*^11^	*U*^22^	*U*^33^	*U*^12^	*U*^13^	*U*^23^
C1	0.0372 (15)	0.0423 (14)	0.0285 (13)	−0.0010 (12)	0.0124 (11)	−0.0022 (11)
C2	0.0447 (17)	0.0600 (18)	0.0416 (16)	0.0010 (14)	0.0240 (14)	0.0026 (13)
C5	0.0452 (17)	0.0385 (14)	0.0416 (15)	0.0057 (13)	0.0193 (13)	0.0040 (12)
C6	0.0349 (14)	0.0371 (14)	0.0302 (13)	−0.0036 (11)	0.0125 (11)	−0.0062 (11)
C7	0.0367 (15)	0.0371 (14)	0.0297 (12)	−0.0024 (12)	0.0145 (11)	−0.0032 (11)
C8	0.093 (3)	0.120 (4)	0.166 (5)	−0.062 (3)	0.099 (4)	−0.071 (4)
C9	0.071 (3)	0.121 (3)	0.061 (2)	−0.016 (2)	0.046 (2)	−0.024 (2)
C10	0.067 (2)	0.070 (2)	0.0382 (17)	0.0162 (19)	−0.0001 (16)	0.0003 (15)
C11	0.078 (3)	0.0420 (18)	0.060 (2)	−0.0011 (17)	0.0206 (19)	0.0045 (16)
N1	0.0393 (13)	0.0379 (12)	0.0330 (11)	−0.0016 (10)	0.0193 (10)	0.0002 (9)
N2	0.0422 (15)	0.0462 (14)	0.0355 (13)	0.0056 (12)	0.0176 (11)	0.0022 (11)
S1	0.0427 (4)	0.0504 (4)	0.0366 (3)	0.0040 (4)	0.0197 (3)	0.0082 (3)
C12	0.0346 (14)	0.0440 (15)	0.0286 (12)	−0.0072 (12)	0.0138 (11)	−0.0075 (11)
C13	0.0291 (14)	0.0486 (16)	0.0385 (15)	−0.0007 (12)	0.0116 (12)	−0.0027 (12)
C14	0.0293 (15)	0.0632 (19)	0.0391 (15)	−0.0007 (14)	0.0171 (13)	−0.0070 (14)
C15	0.0437 (17)	0.0619 (19)	0.0347 (14)	−0.0018 (15)	0.0190 (13)	−0.0020 (14)
C16	0.065 (2)	0.0589 (19)	0.0413 (17)	0.0144 (17)	0.0273 (16)	0.0111 (14)
C17	0.0539 (19)	0.0529 (18)	0.0432 (17)	0.0113 (15)	0.0276 (15)	0.0007 (14)
C18	0.0381 (15)	0.0422 (15)	0.0364 (14)	−0.0066 (13)	0.0173 (12)	−0.0057 (12)
C19	0.075 (2)	0.095 (3)	0.0514 (19)	0.017 (2)	0.0410 (18)	0.0169 (18)
O1	0.0653 (14)	0.0525 (12)	0.0554 (13)	0.0081 (10)	0.0429 (11)	0.0050 (10)
O2	0.0452 (11)	0.0479 (11)	0.0386 (10)	−0.0012 (9)	0.0203 (9)	0.0012 (9)

### 2-Amino-4,4,7,7-tetramethyl-3a,5,6,7a-tetrahydrobenzothiazol-3-ium 4-methylbenzoate (I) Geometric parameters (Å, º)

**Table:**

C1—C6	1.338 (4)	C9—H9A	0.9800
C1—C2	1.502 (4)	C9—H9B	0.9800
C1—S1	1.753 (3)	C9—H9C	0.9800
C2—C8	1.509 (5)	C10—H10A	0.9800
C2—C3B	1.514 (6)	C10—H10B	0.9800
C2—C9	1.518 (5)	C10—H10C	0.9800
C2—C3A	1.626 (9)	C11—H11A	0.9800
C3A—C4A	1.451 (14)	C11—H11B	0.9800
C3A—H3A1	0.9900	C11—H11C	0.9800
C3A—H3A2	0.9900	N1—H1N	0.88 (3)
C4A—C5	1.555 (9)	N2—H2N	0.83 (3)
C4A—H4A1	0.9900	N2—H3N	0.89 (3)
C4A—H4A2	0.9900	C12—C17	1.385 (4)
C3B—C4B	1.551 (9)	C12—C13	1.389 (4)
C3B—H3B1	0.9900	C12—C18	1.506 (4)
C3B—H3B2	0.9900	C13—C14	1.389 (4)
C4B—C5	1.547 (6)	C13—H13	0.9500
C4B—H4B1	0.9900	C14—C15	1.377 (4)
C4B—H4B2	0.9900	C14—H14	0.9500
C5—C6	1.514 (4)	C15—C16	1.386 (4)
C5—C11	1.524 (4)	C15—C19	1.510 (4)
C5—C10	1.529 (4)	C16—C17	1.383 (4)
C6—N1	1.394 (4)	C16—H16	0.9500
C7—N2	1.322 (4)	C17—H17	0.9500
C7—N1	1.325 (3)	C18—O1	1.252 (3)
C7—S1	1.729 (3)	C18—O2	1.267 (3)
C8—H8A	0.9800	C19—H19A	0.9800
C8—H8B	0.9800	C19—H19B	0.9800
C8—H8C	0.9800	C19—H19C	0.9800
			
C6—C1—C2	127.0 (3)	H8A—C8—H8C	109.5
C6—C1—S1	110.6 (2)	H8B—C8—H8C	109.5
C2—C1—S1	122.4 (2)	C2—C9—H9A	109.5
C1—C2—C8	111.6 (3)	C2—C9—H9B	109.5
C1—C2—C3B	105.9 (3)	H9A—C9—H9B	109.5
C8—C2—C3B	98.4 (4)	C2—C9—H9C	109.5
C1—C2—C9	110.6 (3)	H9A—C9—H9C	109.5
C8—C2—C9	108.5 (3)	H9B—C9—H9C	109.5
C3B—C2—C9	121.2 (4)	C5—C10—H10A	109.5
C1—C2—C3A	103.1 (4)	C5—C10—H10B	109.5
C8—C2—C3A	128.8 (5)	H10A—C10—H10B	109.5
C9—C2—C3A	92.3 (4)	C5—C10—H10C	109.5
C4A—C3A—C2	114.0 (8)	H10A—C10—H10C	109.5
C4A—C3A—H3A1	108.8	H10B—C10—H10C	109.5
C2—C3A—H3A1	108.8	C5—C11—H11A	109.5
C4A—C3A—H3A2	108.8	C5—C11—H11B	109.5
C2—C3A—H3A2	108.8	H11A—C11—H11B	109.5
H3A1—C3A—H3A2	107.7	C5—C11—H11C	109.5
C3A—C4A—C5	110.6 (8)	H11A—C11—H11C	109.5
C3A—C4A—H4A1	109.5	H11B—C11—H11C	109.5
C5—C4A—H4A1	109.5	C7—N1—C6	114.1 (2)
C3A—C4A—H4A2	109.5	C7—N1—H1N	116 (2)
C5—C4A—H4A2	109.5	C6—N1—H1N	130.1 (19)
H4A1—C4A—H4A2	108.1	C7—N2—H2N	116 (2)
C2—C3B—C4B	111.3 (5)	C7—N2—H3N	115 (2)
C2—C3B—H3B1	109.4	H2N—N2—H3N	126 (3)
C4B—C3B—H3B1	109.4	C7—S1—C1	90.27 (13)
C2—C3B—H3B2	109.4	C17—C12—C13	118.4 (3)
C4B—C3B—H3B2	109.4	C17—C12—C18	120.5 (3)
H3B1—C3B—H3B2	108.0	C13—C12—C18	121.1 (3)
C5—C4B—C3B	113.0 (5)	C14—C13—C12	120.2 (3)
C5—C4B—H4B1	109.0	C14—C13—H13	119.9
C3B—C4B—H4B1	109.0	C12—C13—H13	119.9
C5—C4B—H4B2	109.0	C15—C14—C13	121.6 (3)
C3B—C4B—H4B2	109.0	C15—C14—H14	119.2
H4B1—C4B—H4B2	107.8	C13—C14—H14	119.2
C6—C5—C11	109.8 (2)	C14—C15—C16	117.9 (3)
C6—C5—C10	109.8 (2)	C14—C15—C19	121.2 (3)
C11—C5—C10	108.7 (3)	C16—C15—C19	120.8 (3)
C6—C5—C4B	106.5 (3)	C17—C16—C15	121.1 (3)
C11—C5—C4B	103.1 (4)	C17—C16—H16	119.4
C10—C5—C4B	118.6 (4)	C15—C16—H16	119.4
C6—C5—C4A	107.9 (4)	C16—C17—C12	120.8 (3)
C11—C5—C4A	121.6 (5)	C16—C17—H17	119.6
C10—C5—C4A	98.1 (5)	C12—C17—H17	119.6
C1—C6—N1	113.5 (2)	O1—C18—O2	124.9 (2)
C1—C6—C5	125.6 (3)	O1—C18—C12	117.2 (3)
N1—C6—C5	121.0 (2)	O2—C18—C12	117.9 (2)
N2—C7—N1	124.6 (2)	C15—C19—H19A	109.5
N2—C7—S1	123.9 (2)	C15—C19—H19B	109.5
N1—C7—S1	111.6 (2)	H19A—C19—H19B	109.5
C2—C8—H8A	109.5	C15—C19—H19C	109.5
C2—C8—H8B	109.5	H19A—C19—H19C	109.5
H8A—C8—H8B	109.5	H19B—C19—H19C	109.5
C2—C8—H8C	109.5		

### 2-Amino-4,4,7,7-tetramethyl-3a,5,6,7a-tetrahydrobenzothiazol-3-ium 4-methylbenzoate (I) Hydrogen-bond geometry (Å, º)

**Table:**

*D*—H···*A*	*D*—H	H···*A*	*D*···*A*	*D*—H···*A*
N1—H1*N*···O1	0.88 (3)	1.76 (3)	2.632 (3)	175 (3)
N2—H2*N*···O2	0.83 (3)	1.98 (4)	2.801 (3)	170 (3)
N2—H3*N*···O2^i^	0.89 (3)	1.93 (3)	2.805 (3)	164 (3)

Symmetry code: (i) *x*, −*y*+1, *z*+1/2.

### 2-Amino-4,4,7,7-tetramethyl-4,5,6,7-tetrahydro-1,3-benzothiazol-3-ium 4-bromobenzoate (II) Crystal data

**Table:**

C_11_H_19_N_2_S^+^·C_7_H_4_BrO_2_^−^	*F*(000) = 424
*M**_r_* = 411.35	*D*_x_ = 1.424 Mg m^−^^3^
Monoclinic, *P**c*	Mo *K*α radiation, λ = 0.71073 Å
*a* = 10.2506 (5) Å	Cell parameters from 6835 reflections
*b* = 11.5855 (4) Å	θ = 2.2–28.2°
*c* = 8.8269 (4) Å	µ = 2.26 mm^−^^1^
β = 113.807 (6)°	*T* = 173 K
*V* = 959.07 (8) Å^3^	Plate, colourless
*Z* = 2	0.12 × 0.08 × 0.02 mm

### 2-Amino-4,4,7,7-tetramethyl-4,5,6,7-tetrahydro-1,3-benzothiazol-3-ium 4-bromobenzoate (II) Data collection

**Table:**

Rigaku XtaLAB P200 HPC diffractometer	3993 independent reflections
Radiation source: rotating anode, Rigaku FR-X	3311 reflections with *I* > 2σ(*I*)
Rigaku Osmic Confocal Optical System monochromator	*R*_int_ = 0.026
ω scans	θ_max_ = 29.1°, θ_min_ = 2.2°
Absorption correction: multi-scan (CrysAlis PRO; Rigaku, 2017)	*h* = −13→13
*T*_min_ = 0.761, *T*_max_ = 1.000	*k* = −15→15
12220 measured reflections	*l* = −10→11

### 2-Amino-4,4,7,7-tetramethyl-4,5,6,7-tetrahydro-1,3-benzothiazol-3-ium 4-bromobenzoate (II) Refinement

**Table:**

Refinement on *F*^2^	Hydrogen site location: mixed
Least-squares matrix: full	H atoms treated by a mixture of independent and constrained refinement
*R*[*F*^2^ > 2σ(*F*^2^)] = 0.032	*w* = 1/[σ^2^(*F*_o_^2^) + (0.0407*P*)^2^ + 0.0111*P*] where *P* = (*F*_o_^2^ + 2*F*_c_^2^)/3
*wR*(*F*^2^) = 0.083	(Δ/σ)_max_ = 0.001
*S* = 1.06	Δρ_max_ = 0.62 e Å^−^^3^
3993 reflections	Δρ_min_ = −0.38 e Å^−^^3^
230 parameters	Absolute structure: Flack *x* determined using 1344 quotients [(*I*^+^)-(*I*^-^)]/[(*I*^+^)+(*I*^-^)] (Parsons *et al.*, 2013).
2 restraints	Absolute structure parameter: 0.005 (4)
Primary atom site location: structure-invariant direct methods	

### 2-Amino-4,4,7,7-tetramethyl-4,5,6,7-tetrahydro-1,3-benzothiazol-3-ium 4-bromobenzoate (II) Special details

**Table:**

Geometry. All esds (except the esd in the dihedral angle between two l.s. planes) are estimated using the full covariance matrix. The cell esds are taken into account individually in the estimation of esds in distances, angles and torsion angles; correlations between esds in cell parameters are only used when they are defined by crystal symmetry. An approximate (isotropic) treatment of cell esds is used for estimating esds involving l.s. planes.

### 2-Amino-4,4,7,7-tetramethyl-4,5,6,7-tetrahydro-1,3-benzothiazol-3-ium 4-bromobenzoate (II) Fractional atomic coordinates and isotropic or equivalent isotropic displacement parameters (Å^2^)

**Table:**

	*x*	*y*	*z*	*U*_iso_*/*U*_eq_	
C1	0.6357 (4)	0.6587 (3)	0.1418 (5)	0.0359 (8)	
C2	0.7655 (5)	0.6679 (4)	0.1027 (5)	0.0446 (9)	
C3	0.8711 (8)	0.7399 (8)	0.2385 (10)	0.113 (3)	
H3A	0.9213	0.6883	0.3338	0.136*	
H3B	0.9431	0.7681	0.1991	0.136*	
C4	0.8218 (6)	0.8359 (5)	0.2982 (9)	0.0780 (17)	
H4A	0.7931	0.8956	0.2105	0.094*	
H4B	0.9040	0.8673	0.3937	0.094*	
C5	0.6976 (4)	0.8213 (3)	0.3532 (5)	0.0403 (9)	
C6	0.6067 (4)	0.7238 (3)	0.2502 (5)	0.0339 (8)	
C7	0.4084 (4)	0.6086 (3)	0.1598 (5)	0.0345 (8)	
C8	0.8389 (8)	0.5492 (7)	0.1190 (12)	0.110 (3)	
H8A	0.9299	0.5590	0.1087	0.164*	
H8B	0.7771	0.4976	0.0314	0.164*	
H8C	0.8561	0.5156	0.2274	0.164*	
C9	0.7278 (7)	0.7092 (6)	−0.0719 (7)	0.0772 (17)	
H9A	0.8147	0.7152	−0.0923	0.116*	
H9B	0.6820	0.7850	−0.0868	0.116*	
H9C	0.6622	0.6541	−0.1501	0.116*	
C10	0.7484 (6)	0.7930 (5)	0.5371 (6)	0.0664 (14)	
H10A	0.8122	0.8542	0.6028	0.100*	
H10B	0.7995	0.7193	0.5604	0.100*	
H10C	0.6659	0.7875	0.5663	0.100*	
C11	0.6095 (6)	0.9320 (4)	0.3150 (6)	0.0578 (12)	
H11A	0.5714	0.9473	0.1957	0.087*	
H11B	0.6699	0.9966	0.3751	0.087*	
H11C	0.5304	0.9231	0.3496	0.087*	
N1	0.4781 (3)	0.6939 (3)	0.2604 (4)	0.0348 (7)	
H1N	0.441 (5)	0.732 (4)	0.339 (5)	0.042*	
N2	0.2855 (4)	0.5667 (3)	0.1482 (5)	0.0408 (8)	
H2N	0.257 (6)	0.582 (4)	0.208 (6)	0.049*	
H3N	0.264 (5)	0.505 (4)	0.086 (6)	0.049*	
S1	0.49893 (11)	0.55873 (9)	0.04481 (11)	0.0398 (2)	
C12	0.2601 (4)	0.7536 (3)	0.6483 (4)	0.0352 (8)	
C13	0.1566 (4)	0.6980 (3)	0.6846 (5)	0.0369 (8)	
H13	0.1094	0.6326	0.6213	0.044*	
C14	0.1208 (4)	0.7360 (4)	0.8116 (5)	0.0422 (9)	
H14	0.0501	0.6970	0.8361	0.051*	
C15	0.1893 (4)	0.8310 (4)	0.9017 (5)	0.0402 (9)	
C16	0.2946 (5)	0.8860 (3)	0.8709 (6)	0.0490 (11)	
H16	0.3434	0.9499	0.9369	0.059*	
C17	0.3291 (5)	0.8481 (4)	0.7443 (5)	0.0466 (10)	
H17	0.4009	0.8871	0.7220	0.056*	
C18	0.2979 (4)	0.7141 (3)	0.5095 (5)	0.0370 (8)	
O1	0.3836 (4)	0.7755 (3)	0.4748 (4)	0.0505 (7)	
O2	0.2410 (3)	0.6225 (2)	0.4343 (4)	0.0419 (6)	
Br1	0.13945 (5)	0.88476 (4)	1.07398 (5)	0.0676 (2)	

### 2-Amino-4,4,7,7-tetramethyl-4,5,6,7-tetrahydro-1,3-benzothiazol-3-ium 4-bromobenzoate (II) Atomic displacement parameters (Å^2^)

**Table:**

	*U*^11^	*U*^22^	*U*^33^	*U*^12^	*U*^13^	*U*^23^
C1	0.037 (2)	0.0392 (18)	0.0320 (18)	0.0012 (17)	0.0138 (17)	0.0030 (16)
C2	0.043 (2)	0.057 (2)	0.040 (2)	−0.004 (2)	0.0238 (19)	−0.0023 (19)
C3	0.087 (5)	0.160 (7)	0.120 (6)	−0.057 (5)	0.071 (5)	−0.073 (6)
C4	0.061 (3)	0.080 (3)	0.109 (5)	−0.032 (3)	0.050 (3)	−0.039 (3)
C5	0.037 (2)	0.0370 (19)	0.043 (2)	−0.0060 (17)	0.0129 (17)	−0.0009 (17)
C6	0.036 (2)	0.0329 (18)	0.0323 (18)	0.0019 (16)	0.0131 (16)	0.0038 (15)
C7	0.037 (2)	0.0365 (19)	0.0293 (18)	0.0022 (17)	0.0123 (16)	0.0043 (16)
C8	0.091 (5)	0.109 (5)	0.167 (8)	0.055 (5)	0.092 (6)	0.064 (5)
C9	0.074 (4)	0.113 (4)	0.058 (3)	0.020 (3)	0.040 (3)	0.028 (3)
C10	0.071 (3)	0.066 (3)	0.039 (2)	−0.016 (3)	−0.002 (2)	−0.001 (2)
C11	0.072 (3)	0.037 (2)	0.059 (3)	0.003 (2)	0.022 (3)	−0.001 (2)
N1	0.0359 (18)	0.0375 (16)	0.0333 (17)	0.0013 (14)	0.0163 (14)	−0.0001 (14)
N2	0.042 (2)	0.0461 (19)	0.037 (2)	−0.0047 (17)	0.0190 (16)	−0.0057 (16)
S1	0.0407 (5)	0.0451 (5)	0.0352 (5)	−0.0029 (5)	0.0168 (4)	−0.0061 (4)
C12	0.034 (2)	0.0390 (19)	0.0305 (19)	0.0064 (16)	0.0109 (16)	0.0081 (16)
C13	0.030 (2)	0.0416 (19)	0.036 (2)	−0.0015 (16)	0.0102 (16)	−0.0011 (16)
C14	0.030 (2)	0.056 (2)	0.041 (2)	0.0004 (19)	0.0153 (18)	0.0030 (19)
C15	0.042 (2)	0.050 (2)	0.0308 (19)	0.0041 (19)	0.0170 (18)	0.0051 (18)
C16	0.060 (3)	0.050 (2)	0.039 (2)	−0.012 (2)	0.023 (2)	−0.0053 (19)
C17	0.055 (3)	0.049 (2)	0.042 (2)	−0.010 (2)	0.026 (2)	0.0014 (19)
C18	0.036 (2)	0.040 (2)	0.035 (2)	0.0061 (18)	0.0153 (17)	0.0025 (18)
O1	0.060 (2)	0.0519 (16)	0.0526 (18)	−0.0091 (15)	0.0365 (16)	−0.0072 (14)
O2	0.0422 (16)	0.0477 (16)	0.0367 (15)	0.0025 (13)	0.0168 (12)	−0.0012 (12)
Br1	0.0863 (4)	0.0796 (3)	0.0542 (3)	−0.0123 (3)	0.0462 (3)	−0.0134 (3)

### 2-Amino-4,4,7,7-tetramethyl-4,5,6,7-tetrahydro-1,3-benzothiazol-3-ium 4-bromobenzoate (II) Geometric parameters (Å, º)

**Table:**

C1—C6	1.342 (5)	C9—H9C	0.9800
C1—C2	1.508 (6)	C10—H10A	0.9800
C1—S1	1.752 (4)	C10—H10B	0.9800
C2—C3	1.503 (8)	C10—H10C	0.9800
C2—C9	1.507 (7)	C11—H11A	0.9800
C2—C8	1.546 (8)	C11—H11B	0.9800
C3—C4	1.408 (10)	C11—H11C	0.9800
C3—H3A	0.9900	N1—H1N	1.01 (5)
C3—H3B	0.9900	N2—H2N	0.72 (5)
C4—C5	1.544 (7)	N2—H3N	0.88 (5)
C4—H4A	0.9900	C12—C13	1.385 (5)
C4—H4B	0.9900	C12—C17	1.391 (6)
C5—C6	1.513 (6)	C12—C18	1.498 (5)
C5—C11	1.526 (6)	C13—C14	1.385 (5)
C5—C10	1.528 (6)	C13—H13	0.9500
C6—N1	1.400 (5)	C14—C15	1.374 (6)
C7—N2	1.316 (5)	C14—H14	0.9500
C7—N1	1.327 (5)	C15—C16	1.371 (6)
C7—S1	1.728 (4)	C15—Br1	1.895 (4)
C8—H8A	0.9800	C16—C17	1.373 (7)
C8—H8B	0.9800	C16—H16	0.9500
C8—H8C	0.9800	C17—H17	0.9500
C9—H9A	0.9800	C18—O1	1.260 (5)
C9—H9B	0.9800	C18—O2	1.263 (5)
			
C6—C1—C2	126.4 (4)	H9A—C9—H9C	109.5
C6—C1—S1	110.8 (3)	H9B—C9—H9C	109.5
C2—C1—S1	122.7 (3)	C5—C10—H10A	109.5
C3—C2—C9	116.4 (5)	C5—C10—H10B	109.5
C3—C2—C1	105.9 (4)	H10A—C10—H10B	109.5
C9—C2—C1	111.8 (4)	C5—C10—H10C	109.5
C3—C2—C8	104.7 (6)	H10A—C10—H10C	109.5
C9—C2—C8	107.0 (5)	H10B—C10—H10C	109.5
C1—C2—C8	110.9 (4)	C5—C11—H11A	109.5
C4—C3—C2	119.0 (6)	C5—C11—H11B	109.5
C4—C3—H3A	107.6	H11A—C11—H11B	109.5
C2—C3—H3A	107.6	C5—C11—H11C	109.5
C4—C3—H3B	107.6	H11A—C11—H11C	109.5
C2—C3—H3B	107.6	H11B—C11—H11C	109.5
H3A—C3—H3B	107.0	C7—N1—C6	114.0 (3)
C3—C4—C5	119.8 (5)	C7—N1—H1N	122 (2)
C3—C4—H4A	107.4	C6—N1—H1N	124 (2)
C5—C4—H4A	107.4	C7—N2—H2N	121 (4)
C3—C4—H4B	107.4	C7—N2—H3N	110 (3)
C5—C4—H4B	107.4	H2N—N2—H3N	126 (5)
H4A—C4—H4B	106.9	C7—S1—C1	90.29 (18)
C6—C5—C11	109.2 (3)	C13—C12—C17	118.3 (4)
C6—C5—C10	110.0 (3)	C13—C12—C18	121.5 (3)
C11—C5—C10	109.4 (4)	C17—C12—C18	120.2 (4)
C6—C5—C4	106.2 (4)	C14—C13—C12	121.2 (4)
C11—C5—C4	109.2 (4)	C14—C13—H13	119.4
C10—C5—C4	112.8 (5)	C12—C13—H13	119.4
C1—C6—N1	113.1 (3)	C15—C14—C13	118.9 (4)
C1—C6—C5	126.0 (3)	C15—C14—H14	120.6
N1—C6—C5	120.9 (3)	C13—C14—H14	120.6
N2—C7—N1	124.5 (4)	C16—C15—C14	121.2 (4)
N2—C7—S1	123.7 (3)	C16—C15—Br1	119.7 (3)
N1—C7—S1	111.7 (3)	C14—C15—Br1	119.2 (3)
C2—C8—H8A	109.5	C15—C16—C17	119.6 (4)
C2—C8—H8B	109.5	C15—C16—H16	120.2
H8A—C8—H8B	109.5	C17—C16—H16	120.2
C2—C8—H8C	109.5	C16—C17—C12	120.9 (4)
H8A—C8—H8C	109.5	C16—C17—H17	119.5
H8B—C8—H8C	109.5	C12—C17—H17	119.5
C2—C9—H9A	109.5	O1—C18—O2	124.5 (4)
C2—C9—H9B	109.5	O1—C18—C12	117.5 (4)
H9A—C9—H9B	109.5	O2—C18—C12	118.0 (3)
C2—C9—H9C	109.5		

### 2-Amino-4,4,7,7-tetramethyl-4,5,6,7-tetrahydro-1,3-benzothiazol-3-ium 4-bromobenzoate (II) Hydrogen-bond geometry (Å, º)

**Table:**

*D*—H···*A*	*D*—H	H···*A*	*D*···*A*	*D*—H···*A*
N1—H1*N*···O1	1.01 (5)	1.62 (5)	2.626 (4)	172 (4)
N2—H2*N*···O2	0.72 (5)	2.12 (5)	2.816 (5)	162 (6)
N2—H3*N*···O2^i^	0.88 (5)	1.94 (5)	2.808 (5)	170 (4)

Symmetry code: (i) *x*, −*y*+1, *z*−1/2.

### 2-Amino-4,4,7,7-tetramethyl-4,5,6,7-tetrahydro-1,3-benzothiazol-3-ium 3,5-dinitrobenzoate (III) Crystal data

**Table:**

C_11_H_19_N_2_S^+^·C_7_H_3_N_2_O_6_^−^	*F*(000) = 888
*M**_r_* = 422.45	*D*_x_ = 1.398 Mg m^−^^3^
Monoclinic, *P*2_1_/*n*	Mo *K*α radiation, λ = 0.71073 Å
*a* = 17.3232 (6) Å	Cell parameters from 10667 reflections
*b* = 5.84087 (17) Å	θ = 2.0–27.8°
*c* = 21.0876 (8) Å	µ = 0.21 mm^−^^1^
β = 109.875 (4)°	*T* = 173 K
*V* = 2006.60 (12) Å^3^	Plate, colourless
*Z* = 4	0.27 × 0.10 × 0.03 mm

### 2-Amino-4,4,7,7-tetramethyl-4,5,6,7-tetrahydro-1,3-benzothiazol-3-ium 3,5-dinitrobenzoate (III) Data collection

**Table:**

Rigaku XtaLAB P200 HPC diffractometer	4698 independent reflections
Radiation source: rotating anode, Rigaku FR-X	3712 reflections with *I* > 2σ(*I*)
Rigaku Osmic Confocal Optical System monochromator	*R*_int_ = 0.056
ω scans	θ_max_ = 29.2°, θ_min_ = 1.9°
Absorption correction: multi-scan (CrysAlis PRO; Rigaku, 2017)	*h* = −21→23
*T*_min_ = 0.566, *T*_max_ = 1.000	*k* = −7→7
25034 measured reflections	*l* = −26→26

### 2-Amino-4,4,7,7-tetramethyl-4,5,6,7-tetrahydro-1,3-benzothiazol-3-ium 3,5-dinitrobenzoate (III) Refinement

**Table:**

Refinement on *F*^2^	Primary atom site location: structure-invariant direct methods
Least-squares matrix: full	Hydrogen site location: mixed
*R*[*F*^2^ > 2σ(*F*^2^)] = 0.042	H atoms treated by a mixture of independent and constrained refinement
*wR*(*F*^2^) = 0.116	*w* = 1/[σ^2^(*F*_o_^2^) + (0.0567*P*)^2^ + 0.5151*P*] where *P* = (*F*_o_^2^ + 2*F*_c_^2^)/3
*S* = 1.05	(Δ/σ)_max_ < 0.001
4698 reflections	Δρ_max_ = 0.49 e Å^−^^3^
275 parameters	Δρ_min_ = −0.25 e Å^−^^3^
0 restraints	

### 2-Amino-4,4,7,7-tetramethyl-4,5,6,7-tetrahydro-1,3-benzothiazol-3-ium 3,5-dinitrobenzoate (III) Special details

**Table:**

Geometry. All esds (except the esd in the dihedral angle between two l.s. planes) are estimated using the full covariance matrix. The cell esds are taken into account individually in the estimation of esds in distances, angles and torsion angles; correlations between esds in cell parameters are only used when they are defined by crystal symmetry. An approximate (isotropic) treatment of cell esds is used for estimating esds involving l.s. planes.

### 2-Amino-4,4,7,7-tetramethyl-4,5,6,7-tetrahydro-1,3-benzothiazol-3-ium 3,5-dinitrobenzoate (III) Fractional atomic coordinates and isotropic or equivalent isotropic displacement parameters (Å^2^)

**Table:**

	*x*	*y*	*z*	*U*_iso_*/*U*_eq_	
C1	0.26545 (9)	0.4584 (3)	0.49126 (7)	0.0264 (3)	
C2	0.19150 (9)	0.3629 (3)	0.50428 (8)	0.0298 (3)	
C3	0.13800 (10)	0.2456 (3)	0.43879 (9)	0.0419 (4)	
H3A	0.0996	0.1392	0.4494	0.050*	
H3B	0.1048	0.3636	0.4077	0.050*	
C4	0.18675 (11)	0.1131 (3)	0.40303 (9)	0.0397 (4)	
H4A	0.1479	0.0344	0.3634	0.048*	
H4B	0.2196	−0.0059	0.4340	0.048*	
C5	0.24503 (10)	0.2636 (3)	0.37920 (8)	0.0319 (4)	
C6	0.28907 (9)	0.4173 (3)	0.43809 (7)	0.0260 (3)	
C7	0.39559 (9)	0.6469 (3)	0.49858 (7)	0.0266 (3)	
C8	0.21999 (11)	0.1932 (3)	0.56307 (9)	0.0388 (4)	
H8A	0.1720	0.1286	0.5713	0.058*	
H8B	0.2547	0.2727	0.6037	0.058*	
H8C	0.2514	0.0697	0.5518	0.058*	
C9	0.14165 (11)	0.5539 (3)	0.52232 (10)	0.0427 (4)	
H9A	0.0925	0.4881	0.5283	0.064*	
H9B	0.1253	0.6672	0.4859	0.064*	
H9C	0.1753	0.6282	0.5643	0.064*	
C10	0.30657 (12)	0.1076 (3)	0.36271 (10)	0.0442 (4)	
H10A	0.2769	−0.0070	0.3294	0.066*	
H10B	0.3410	0.0304	0.4039	0.066*	
H10C	0.3413	0.1996	0.3443	0.066*	
C11	0.19849 (13)	0.4072 (4)	0.31745 (9)	0.0516 (5)	
H11A	0.1707	0.3058	0.2795	0.077*	
H11B	0.2372	0.5057	0.3054	0.077*	
H11C	0.1577	0.5024	0.3278	0.077*	
N1	0.36224 (8)	0.5279 (2)	0.44229 (6)	0.0271 (3)	
H1N	0.3856 (11)	0.524 (3)	0.4110 (9)	0.033*	
N2	0.46553 (9)	0.7605 (3)	0.51363 (7)	0.0337 (3)	
H2N	0.4887 (12)	0.780 (3)	0.4812 (10)	0.040*	
H3N	0.4791 (12)	0.857 (3)	0.5486 (10)	0.040*	
S1	0.33701 (2)	0.63371 (7)	0.55004 (2)	0.02882 (12)	
C12	0.53091 (9)	0.7205 (3)	0.30278 (7)	0.0283 (3)	
C13	0.58549 (9)	0.8968 (3)	0.30559 (8)	0.0304 (3)	
H13	0.5992	1.0042	0.3416	0.037*	
C14	0.61975 (9)	0.9144 (3)	0.25552 (8)	0.0313 (4)	
C15	0.60233 (9)	0.7633 (3)	0.20218 (8)	0.0327 (4)	
H15	0.6256	0.7796	0.1676	0.039*	
C16	0.54918 (9)	0.5872 (3)	0.20198 (8)	0.0306 (3)	
C17	0.51322 (9)	0.5616 (3)	0.25084 (7)	0.0294 (3)	
H17	0.4770	0.4375	0.2489	0.035*	
C18	0.49110 (10)	0.7013 (3)	0.35667 (8)	0.0312 (3)	
N3	0.67709 (8)	1.1050 (3)	0.25924 (7)	0.0380 (3)	
N4	0.52907 (8)	0.4200 (3)	0.14618 (7)	0.0386 (4)	
O1	0.43858 (8)	0.5479 (2)	0.34910 (6)	0.0427 (3)	
O2	0.51480 (8)	0.8407 (2)	0.40440 (6)	0.0410 (3)	
O3	0.69300 (8)	1.1515 (2)	0.20855 (7)	0.0500 (4)	
O4	0.70535 (9)	1.2066 (3)	0.31259 (7)	0.0596 (4)	
O5	0.49224 (9)	0.2464 (3)	0.15173 (7)	0.0508 (3)	
O6	0.54967 (8)	0.4645 (3)	0.09754 (6)	0.0522 (4)	

### 2-Amino-4,4,7,7-tetramethyl-4,5,6,7-tetrahydro-1,3-benzothiazol-3-ium 3,5-dinitrobenzoate (III) Atomic displacement parameters (Å^2^)

**Table:**

	*U*^11^	*U*^22^	*U*^33^	*U*^12^	*U*^13^	*U*^23^
C1	0.0251 (7)	0.0287 (8)	0.0255 (8)	−0.0025 (6)	0.0086 (6)	−0.0005 (6)
C2	0.0252 (8)	0.0364 (8)	0.0307 (8)	−0.0032 (6)	0.0130 (6)	0.0006 (7)
C3	0.0301 (9)	0.0543 (11)	0.0417 (10)	−0.0134 (8)	0.0129 (7)	−0.0047 (9)
C4	0.0385 (10)	0.0457 (10)	0.0350 (9)	−0.0158 (8)	0.0126 (7)	−0.0077 (8)
C5	0.0335 (8)	0.0374 (9)	0.0250 (8)	−0.0096 (7)	0.0103 (6)	−0.0039 (7)
C6	0.0252 (7)	0.0286 (8)	0.0248 (7)	−0.0034 (6)	0.0094 (6)	0.0005 (6)
C7	0.0290 (8)	0.0282 (8)	0.0250 (8)	−0.0031 (6)	0.0123 (6)	−0.0006 (6)
C8	0.0378 (9)	0.0441 (10)	0.0412 (10)	−0.0014 (8)	0.0222 (8)	0.0061 (8)
C9	0.0329 (9)	0.0470 (10)	0.0535 (11)	0.0034 (8)	0.0215 (8)	0.0005 (9)
C10	0.0509 (11)	0.0441 (10)	0.0437 (10)	−0.0122 (8)	0.0239 (9)	−0.0160 (8)
C11	0.0576 (12)	0.0607 (13)	0.0276 (9)	−0.0091 (10)	0.0029 (8)	0.0015 (9)
N1	0.0296 (7)	0.0320 (7)	0.0236 (6)	−0.0064 (5)	0.0140 (5)	−0.0029 (5)
N2	0.0352 (8)	0.0398 (8)	0.0304 (7)	−0.0140 (6)	0.0168 (6)	−0.0085 (6)
S1	0.0292 (2)	0.0350 (2)	0.0259 (2)	−0.00573 (16)	0.01407 (16)	−0.00554 (15)
C12	0.0239 (7)	0.0392 (9)	0.0224 (7)	0.0017 (6)	0.0088 (6)	0.0036 (6)
C13	0.0273 (8)	0.0396 (9)	0.0254 (8)	−0.0003 (7)	0.0103 (6)	0.0020 (7)
C14	0.0230 (8)	0.0425 (9)	0.0291 (8)	0.0021 (7)	0.0099 (6)	0.0083 (7)
C15	0.0236 (8)	0.0520 (10)	0.0243 (8)	0.0090 (7)	0.0106 (6)	0.0086 (7)
C16	0.0230 (7)	0.0451 (9)	0.0220 (7)	0.0085 (7)	0.0056 (6)	0.0002 (7)
C17	0.0221 (7)	0.0390 (8)	0.0257 (8)	0.0006 (6)	0.0063 (6)	0.0015 (7)
C18	0.0300 (8)	0.0392 (9)	0.0271 (8)	−0.0042 (7)	0.0133 (6)	−0.0005 (7)
N3	0.0305 (7)	0.0491 (9)	0.0396 (8)	−0.0014 (6)	0.0184 (6)	0.0090 (7)
N4	0.0273 (7)	0.0557 (10)	0.0313 (8)	0.0099 (7)	0.0081 (6)	−0.0056 (7)
O1	0.0462 (7)	0.0556 (8)	0.0347 (7)	−0.0218 (6)	0.0244 (6)	−0.0106 (6)
O2	0.0512 (8)	0.0453 (7)	0.0363 (7)	−0.0158 (6)	0.0275 (6)	−0.0114 (5)
O3	0.0466 (8)	0.0666 (9)	0.0461 (8)	−0.0058 (6)	0.0280 (6)	0.0147 (6)
O4	0.0662 (10)	0.0704 (10)	0.0507 (9)	−0.0328 (8)	0.0311 (7)	−0.0119 (7)
O5	0.0504 (8)	0.0563 (9)	0.0448 (8)	−0.0036 (7)	0.0149 (6)	−0.0156 (6)
O6	0.0452 (8)	0.0852 (10)	0.0316 (7)	0.0108 (7)	0.0199 (6)	−0.0082 (7)

### 2-Amino-4,4,7,7-tetramethyl-4,5,6,7-tetrahydro-1,3-benzothiazol-3-ium 3,5-dinitrobenzoate (III) Geometric parameters (Å, º)

**Table:**

C1—C6	1.340 (2)	C10—H10B	0.9800
C1—C2	1.506 (2)	C10—H10C	0.9800
C1—S1	1.7540 (15)	C11—H11A	0.9800
C2—C8	1.532 (2)	C11—H11B	0.9800
C2—C9	1.535 (2)	C11—H11C	0.9800
C2—C3	1.538 (2)	N1—H1N	0.885 (19)
C3—C4	1.521 (3)	N2—H2N	0.91 (2)
C3—H3A	0.9900	N2—H3N	0.90 (2)
C3—H3B	0.9900	C12—C13	1.385 (2)
C4—C5	1.546 (2)	C12—C17	1.388 (2)
C4—H4A	0.9900	C12—C18	1.521 (2)
C4—H4B	0.9900	C13—C14	1.380 (2)
C5—C6	1.512 (2)	C13—H13	0.9500
C5—C11	1.528 (2)	C14—C15	1.380 (2)
C5—C10	1.529 (2)	C14—N3	1.476 (2)
C6—N1	1.3981 (19)	C15—C16	1.380 (2)
C7—N2	1.322 (2)	C15—H15	0.9500
C7—N1	1.3267 (19)	C16—C17	1.381 (2)
C7—S1	1.7211 (15)	C16—N4	1.477 (2)
C8—H8A	0.9800	C17—H17	0.9500
C8—H8B	0.9800	C18—O1	1.2475 (19)
C8—H8C	0.9800	C18—O2	1.2506 (19)
C9—H9A	0.9800	N3—O4	1.2178 (19)
C9—H9B	0.9800	N3—O3	1.2218 (18)
C9—H9C	0.9800	N4—O6	1.2231 (19)
C10—H10A	0.9800	N4—O5	1.224 (2)
			
C6—C1—C2	127.39 (14)	C5—C10—H10B	109.5
C6—C1—S1	110.64 (11)	H10A—C10—H10B	109.5
C2—C1—S1	121.88 (11)	C5—C10—H10C	109.5
C1—C2—C8	109.11 (13)	H10A—C10—H10C	109.5
C1—C2—C9	111.17 (13)	H10B—C10—H10C	109.5
C8—C2—C9	108.85 (13)	C5—C11—H11A	109.5
C1—C2—C3	106.95 (12)	C5—C11—H11B	109.5
C8—C2—C3	111.61 (14)	H11A—C11—H11B	109.5
C9—C2—C3	109.15 (14)	C5—C11—H11C	109.5
C4—C3—C2	113.90 (14)	H11A—C11—H11C	109.5
C4—C3—H3A	108.8	H11B—C11—H11C	109.5
C2—C3—H3A	108.8	C7—N1—C6	114.09 (12)
C4—C3—H3B	108.8	C7—N1—H1N	120.8 (12)
C2—C3—H3B	108.8	C6—N1—H1N	125.1 (12)
H3A—C3—H3B	107.7	C7—N2—H2N	119.0 (12)
C3—C4—C5	113.96 (15)	C7—N2—H3N	118.5 (12)
C3—C4—H4A	108.8	H2N—N2—H3N	118.8 (17)
C5—C4—H4A	108.8	C7—S1—C1	90.40 (7)
C3—C4—H4B	108.8	C13—C12—C17	119.67 (14)
C5—C4—H4B	108.8	C13—C12—C18	119.95 (14)
H4A—C4—H4B	107.7	C17—C12—C18	120.38 (14)
C6—C5—C11	110.26 (14)	C14—C13—C12	119.18 (15)
C6—C5—C10	110.14 (13)	C14—C13—H13	120.4
C11—C5—C10	109.86 (15)	C12—C13—H13	120.4
C6—C5—C4	105.88 (12)	C13—C14—C15	122.97 (15)
C11—C5—C4	112.06 (15)	C13—C14—N3	118.18 (15)
C10—C5—C4	108.56 (14)	C15—C14—N3	118.85 (14)
C1—C6—N1	113.12 (13)	C16—C15—C14	116.12 (14)
C1—C6—C5	125.55 (13)	C16—C15—H15	121.9
N1—C6—C5	121.31 (12)	C14—C15—H15	121.9
N2—C7—N1	124.23 (14)	C15—C16—C17	123.23 (15)
N2—C7—S1	124.04 (12)	C15—C16—N4	118.16 (14)
N1—C7—S1	111.72 (11)	C17—C16—N4	118.60 (15)
C2—C8—H8A	109.5	C16—C17—C12	118.79 (15)
C2—C8—H8B	109.5	C16—C17—H17	120.6
H8A—C8—H8B	109.5	C12—C17—H17	120.6
C2—C8—H8C	109.5	O1—C18—O2	126.60 (14)
H8A—C8—H8C	109.5	O1—C18—C12	116.98 (14)
H8B—C8—H8C	109.5	O2—C18—C12	116.42 (13)
C2—C9—H9A	109.5	O4—N3—O3	124.09 (15)
C2—C9—H9B	109.5	O4—N3—C14	117.95 (13)
H9A—C9—H9B	109.5	O3—N3—C14	117.96 (15)
C2—C9—H9C	109.5	O6—N4—O5	124.50 (15)
H9A—C9—H9C	109.5	O6—N4—C16	118.07 (16)
H9B—C9—H9C	109.5	O5—N4—C16	117.43 (14)
C5—C10—H10A	109.5		
			
C6—C1—C2—C8	109.98 (18)	N1—C7—S1—C1	0.11 (12)
S1—C1—C2—C8	−66.27 (17)	C6—C1—S1—C7	0.86 (12)
C6—C1—C2—C9	−129.96 (17)	C2—C1—S1—C7	177.68 (13)
S1—C1—C2—C9	53.79 (18)	C17—C12—C13—C14	1.7 (2)
C6—C1—C2—C3	−10.9 (2)	C18—C12—C13—C14	−178.67 (14)
S1—C1—C2—C3	172.86 (12)	C12—C13—C14—C15	−0.1 (2)
C1—C2—C3—C4	40.0 (2)	C12—C13—C14—N3	179.39 (13)
C8—C2—C3—C4	−79.31 (18)	C13—C14—C15—C16	−1.3 (2)
C9—C2—C3—C4	160.33 (15)	N3—C14—C15—C16	179.18 (13)
C2—C3—C4—C5	−62.4 (2)	C14—C15—C16—C17	1.3 (2)
C3—C4—C5—C6	46.04 (19)	C14—C15—C16—N4	−179.58 (13)
C3—C4—C5—C11	−74.21 (19)	C15—C16—C17—C12	0.2 (2)
C3—C4—C5—C10	164.27 (15)	N4—C16—C17—C12	−178.97 (14)
C2—C1—C6—N1	−178.20 (14)	C13—C12—C17—C16	−1.7 (2)
S1—C1—C6—N1	−1.60 (17)	C18—C12—C17—C16	178.65 (14)
C2—C1—C6—C5	0.1 (3)	C13—C12—C18—O1	175.54 (15)
S1—C1—C6—C5	176.71 (13)	C17—C12—C18—O1	−4.8 (2)
C11—C5—C6—C1	104.46 (19)	C13—C12—C18—O2	−4.8 (2)
C10—C5—C6—C1	−134.13 (17)	C17—C12—C18—O2	174.86 (15)
C4—C5—C6—C1	−16.9 (2)	C13—C14—N3—O4	15.8 (2)
C11—C5—C6—N1	−77.36 (19)	C15—C14—N3—O4	−164.70 (16)
C10—C5—C6—N1	44.0 (2)	C13—C14—N3—O3	−164.01 (15)
C4—C5—C6—N1	161.23 (14)	C15—C14—N3—O3	15.5 (2)
N2—C7—N1—C6	178.54 (15)	C15—C16—N4—O6	−11.4 (2)
S1—C7—N1—C6	−1.05 (17)	C17—C16—N4—O6	167.84 (14)
C1—C6—N1—C7	1.76 (19)	C15—C16—N4—O5	169.17 (14)
C5—C6—N1—C7	−176.63 (14)	C17—C16—N4—O5	−11.6 (2)
N2—C7—S1—C1	−179.48 (15)		

### 2-Amino-4,4,7,7-tetramethyl-4,5,6,7-tetrahydro-1,3-benzothiazol-3-ium 3,5-dinitrobenzoate (III) Hydrogen-bond geometry (Å, º)

**Table:**

*D*—H···*A*	*D*—H	H···*A*	*D*···*A*	*D*—H···*A*
N1—H1*N*···O1	0.885 (19)	1.838 (19)	2.7187 (17)	173.4 (17)
N2—H3*N*···O2^i^	0.90 (2)	2.01 (2)	2.8509 (19)	156.3 (17)
N2—H2*N*···O2	0.91 (2)	1.86 (2)	2.7551 (18)	168.3 (18)
C8—H8*A*···O6^ii^	0.98	2.43	3.397 (2)	170

Symmetry codes: (i) −*x*+1, −*y*+2, −*z*+1; (ii) *x*−1/2, −*y*+1/2, *z*+1/2.

### Bis(2-amino-4,4,7,7-tetramethyl-4,5,6,7-tetrahydro-1,3-benzothiazol-3-ium) fumarate (IV) Crystal data

**Table:**

2C_11_H_19_N_2_S^+^·C_4_H_2_O_4_^2^^−^	*F*(000) = 576
*M**_r_* = 536.74	*D*_x_ = 1.224 Mg m^−^^3^
Monoclinic, *P*2_1_	Mo *K*α radiation, λ = 0.71073 Å
*a* = 9.0259 (6) Å	Cell parameters from 4569 reflections
*b* = 14.7314 (11) Å	θ = 2.3–23.7°
*c* = 11.1993 (8) Å	µ = 0.22 mm^−^^1^
β = 101.943 (6)°	*T* = 173 K
*V* = 1456.87 (18) Å^3^	Prism, orange
*Z* = 2	0.15 × 0.08 × 0.02 mm

### Bis(2-amino-4,4,7,7-tetramethyl-4,5,6,7-tetrahydro-1,3-benzothiazol-3-ium) fumarate (IV) Data collection

**Table:**

Rigaku XtaLAB P200 HPC diffractometer	6424 independent reflections
Radiation source: rotating anode, Rigaku FR-X	3792 reflections with *I* > 2σ(*I*)
Rigaku Osmic Confocal Optical System monochromator	*R*_int_ = 0.080
ω scans	θ_max_ = 29.1°, θ_min_ = 1.9°
Absorption correction: multi-scan (CrysAlis PRO; Rigaku, 2017)	*h* = −12→11
*T*_min_ = 0.333, *T*_max_ = 1.000	*k* = −18→20
19263 measured reflections	*l* = −14→12

### Bis(2-amino-4,4,7,7-tetramethyl-4,5,6,7-tetrahydro-1,3-benzothiazol-3-ium) fumarate (IV) Refinement

**Table:**

Refinement on *F*^2^	Hydrogen site location: mixed
Least-squares matrix: full	H atoms treated by a mixture of independent and constrained refinement
*R*[*F*^2^ > 2σ(*F*^2^)] = 0.062	*w* = 1/[σ^2^(*F*_o_^2^) + (0.0488*P*)^2^] where *P* = (*F*_o_^2^ + 2*F*_c_^2^)/3
*wR*(*F*^2^) = 0.128	(Δ/σ)_max_ < 0.001
*S* = 1.00	Δρ_max_ = 0.27 e Å^−^^3^
6424 reflections	Δρ_min_ = −0.26 e Å^−^^3^
350 parameters	Absolute structure: Flack *x* determined using 1279 quotients [(*I*^+^)-(*I*^-^)]/[(*I*^+^)+(*I*^-^)] (Parsons *et al.*, 2013).
1 restraint	Absolute structure parameter: 0.46 (7)
Primary atom site location: structure-invariant direct methods	

### Bis(2-amino-4,4,7,7-tetramethyl-4,5,6,7-tetrahydro-1,3-benzothiazol-3-ium) fumarate (IV) Special details

**Table:**

Geometry. All esds (except the esd in the dihedral angle between two l.s. planes) are estimated using the full covariance matrix. The cell esds are taken into account individually in the estimation of esds in distances, angles and torsion angles; correlations between esds in cell parameters are only used when they are defined by crystal symmetry. An approximate (isotropic) treatment of cell esds is used for estimating esds involving l.s. planes.

### Bis(2-amino-4,4,7,7-tetramethyl-4,5,6,7-tetrahydro-1,3-benzothiazol-3-ium) fumarate (IV) Fractional atomic coordinates and isotropic or equivalent isotropic displacement parameters (Å^2^)

**Table:**

	*x*	*y*	*z*	*U*_iso_*/*U*_eq_	Occ. (<1)
C1	1.2869 (6)	0.3952 (4)	0.6937 (4)	0.0339 (13)	
C2	1.3939 (6)	0.4406 (4)	0.7989 (5)	0.0467 (15)	
C3A	1.2984 (8)	0.5068 (7)	0.8546 (8)	0.052 (3)*	0.78 (2)
H3A1	1.2720	0.5596	0.7996	0.062*	0.78 (2)
H3A2	1.3578	0.5293	0.9334	0.062*	0.78 (2)
C4A	1.1520 (10)	0.4615 (8)	0.8755 (8)	0.047 (2)*	0.78 (2)
H4A1	1.1017	0.5022	0.9252	0.056*	0.78 (2)
H4A2	1.1780	0.4044	0.9216	0.056*	0.78 (2)
C3B	1.284 (3)	0.456 (3)	0.899 (3)	0.063 (12)*	0.22 (2)
H3B1	1.2677	0.3968	0.9362	0.075*	0.22 (2)
H3B2	1.3377	0.4959	0.9657	0.075*	0.22 (2)
C4B	1.130 (3)	0.498 (2)	0.846 (3)	0.033 (8)*	0.22 (2)
H4B1	1.0735	0.5078	0.9121	0.039*	0.22 (2)
H4B2	1.1445	0.5582	0.8102	0.039*	0.22 (2)
C5	1.0394 (6)	0.4401 (4)	0.7513 (4)	0.0372 (13)	
C6	1.1349 (6)	0.3955 (3)	0.6728 (4)	0.0312 (13)	
C7	1.1687 (6)	0.3114 (3)	0.5087 (4)	0.0320 (13)	
C8	1.4824 (7)	0.3683 (4)	0.8815 (6)	0.067 (2)	
H8A	1.5474	0.3975	0.9519	0.101*	
H8B	1.5451	0.3337	0.8359	0.101*	
H8C	1.4115	0.3272	0.9098	0.101*	
C9	1.5096 (8)	0.4998 (5)	0.7490 (6)	0.076 (2)	
H9A	1.5751	0.5316	0.8168	0.114*	
H9B	1.4553	0.5443	0.6909	0.114*	
H9C	1.5715	0.4610	0.7077	0.114*	
C10	0.9232 (6)	0.3732 (4)	0.7813 (5)	0.0506 (16)	
H10A	0.8700	0.4009	0.8400	0.076*	
H10B	0.9750	0.3180	0.8167	0.076*	
H10C	0.8501	0.3578	0.7065	0.076*	
C11	0.9553 (8)	0.5223 (5)	0.6843 (6)	0.070 (2)	
H11A	0.8941	0.5512	0.7364	0.105*	
H11B	0.8892	0.5020	0.6083	0.105*	
H11C	1.0291	0.5661	0.6656	0.105*	
N1	1.0694 (5)	0.3476 (3)	0.5669 (4)	0.0298 (11)	
H1N	0.976 (6)	0.334 (4)	0.544 (4)	0.036*	
N2	1.1307 (6)	0.2614 (4)	0.4096 (4)	0.0452 (13)	
H2N	1.029 (7)	0.263 (4)	0.369 (5)	0.054*	
H3N	1.202 (6)	0.248 (4)	0.365 (5)	0.054*	
S1	1.35226 (14)	0.33370 (9)	0.58194 (12)	0.0396 (4)	
C12	−0.1633 (6)	0.1107 (4)	−0.1894 (4)	0.0340 (13)	
C13	−0.2578 (6)	0.0698 (4)	−0.3022 (5)	0.0408 (14)	
C14	−0.1503 (6)	0.0460 (4)	−0.3869 (5)	0.0501 (16)	
H14A	−0.2031	0.0048	−0.4519	0.060*	
H14B	−0.1244	0.1022	−0.4265	0.060*	
C15	−0.0046 (6)	0.0005 (4)	−0.3204 (5)	0.0493 (15)	
H15A	0.0557	−0.0170	−0.3813	0.059*	
H15B	−0.0312	−0.0558	−0.2816	0.059*	
C16	0.0948 (6)	0.0599 (4)	−0.2219 (4)	0.0353 (13)	
C17	−0.0114 (5)	0.1088 (3)	−0.1552 (4)	0.0286 (12)	
C18	−0.0619 (6)	0.1945 (4)	0.0014 (5)	0.0338 (13)	
C19	−0.3410 (7)	−0.0144 (4)	−0.2709 (6)	0.0662 (19)	
H19A	−0.4097	−0.0366	−0.3446	0.099*	
H19B	−0.3995	0.0012	−0.2092	0.099*	
H19C	−0.2672	−0.0618	−0.2389	0.099*	
C20	−0.3760 (6)	0.1378 (4)	−0.3673 (6)	0.0556 (17)	
H20A	−0.4356	0.1098	−0.4412	0.083*	
H20B	−0.3246	0.1919	−0.3896	0.083*	
H20C	−0.4432	0.1552	−0.3127	0.083*	
C21	0.2029 (6)	−0.0017 (4)	−0.1334 (5)	0.0498 (15)	
H21A	0.2615	−0.0391	−0.1793	0.075*	
H21B	0.1441	−0.0412	−0.0901	0.075*	
H21C	0.2720	0.0357	−0.0743	0.075*	
C22	0.1855 (7)	0.1290 (4)	−0.2784 (5)	0.0520 (17)	
H22A	0.2544	0.0971	−0.3212	0.078*	
H22B	0.2443	0.1671	−0.2140	0.078*	
H22C	0.1162	0.1671	−0.3364	0.078*	
N3	0.0446 (5)	0.1575 (3)	−0.0477 (4)	0.0329 (11)	
H4N	0.151 (6)	0.163 (4)	−0.013 (4)	0.040*	
N4	−0.0317 (6)	0.2444 (4)	0.1013 (4)	0.0457 (13)	
H5N	0.078 (7)	0.242 (4)	0.141 (5)	0.055*	
H6N	−0.104 (7)	0.256 (4)	0.130 (5)	0.055*	
S2	−0.24072 (14)	0.17401 (9)	−0.08375 (12)	0.0386 (4)	
C23	0.7409 (6)	0.2901 (4)	0.3641 (5)	0.0358 (13)	
C24	0.5781 (6)	0.2738 (4)	0.3122 (5)	0.0392 (14)	
H24	0.5076	0.2871	0.3621	0.047*	
C25	0.5242 (6)	0.2420 (4)	0.2013 (5)	0.0380 (13)	
H25	0.5942	0.2310	0.1502	0.046*	
C26	0.3622 (6)	0.2223 (4)	0.1512 (5)	0.0371 (14)	
O1	0.7763 (4)	0.3234 (3)	0.4689 (3)	0.0469 (10)	
O2	0.8346 (4)	0.2695 (3)	0.2995 (3)	0.0591 (12)	
O3	0.3301 (4)	0.1922 (3)	0.0429 (3)	0.0473 (11)	
O4	0.2689 (4)	0.2349 (4)	0.2157 (3)	0.0642 (14)	

### Bis(2-amino-4,4,7,7-tetramethyl-4,5,6,7-tetrahydro-1,3-benzothiazol-3-ium) fumarate (IV) Atomic displacement parameters (Å^2^)

**Table:**

	*U*^11^	*U*^22^	*U*^33^	*U*^12^	*U*^13^	*U*^23^
C1	0.035 (3)	0.035 (3)	0.031 (3)	0.003 (2)	0.003 (2)	−0.002 (2)
C2	0.040 (3)	0.053 (4)	0.042 (3)	0.004 (3)	−0.001 (3)	−0.011 (3)
C5	0.039 (3)	0.040 (3)	0.033 (3)	0.001 (3)	0.008 (3)	−0.007 (3)
C6	0.041 (3)	0.026 (3)	0.025 (3)	−0.002 (3)	0.003 (2)	−0.003 (2)
C7	0.039 (3)	0.035 (3)	0.022 (3)	−0.004 (2)	0.007 (2)	0.000 (2)
C8	0.064 (4)	0.073 (5)	0.048 (4)	−0.007 (3)	−0.028 (3)	0.007 (3)
C9	0.070 (5)	0.058 (5)	0.090 (5)	−0.021 (4)	−0.006 (4)	−0.004 (4)
C10	0.047 (4)	0.064 (4)	0.047 (4)	−0.003 (3)	0.022 (3)	0.002 (3)
C11	0.085 (5)	0.060 (5)	0.077 (5)	0.025 (4)	0.044 (4)	0.015 (4)
N1	0.030 (2)	0.035 (3)	0.024 (2)	−0.001 (2)	0.006 (2)	0.000 (2)
N2	0.035 (3)	0.064 (4)	0.037 (3)	0.003 (3)	0.007 (2)	−0.013 (3)
S1	0.0327 (7)	0.0473 (9)	0.0377 (8)	0.0049 (7)	0.0045 (6)	−0.0037 (7)
C12	0.039 (3)	0.033 (3)	0.029 (3)	0.001 (3)	0.005 (2)	0.002 (2)
C13	0.038 (3)	0.043 (4)	0.039 (3)	−0.005 (3)	0.001 (3)	0.002 (3)
C14	0.046 (3)	0.066 (4)	0.035 (3)	0.003 (3)	0.000 (3)	−0.018 (3)
C15	0.049 (4)	0.053 (4)	0.043 (3)	−0.002 (3)	0.004 (3)	−0.017 (3)
C16	0.035 (3)	0.041 (3)	0.031 (3)	0.002 (3)	0.007 (2)	−0.005 (3)
C17	0.031 (3)	0.029 (3)	0.024 (3)	0.000 (2)	0.000 (2)	0.003 (2)
C18	0.036 (3)	0.035 (4)	0.030 (3)	0.005 (2)	0.008 (3)	−0.001 (2)
C19	0.068 (4)	0.055 (4)	0.070 (4)	−0.023 (4)	0.001 (4)	−0.004 (3)
C20	0.047 (4)	0.068 (5)	0.048 (4)	0.000 (3)	0.000 (3)	−0.003 (3)
C21	0.054 (4)	0.044 (4)	0.046 (3)	0.016 (3)	0.000 (3)	−0.007 (3)
C22	0.053 (4)	0.064 (5)	0.044 (4)	−0.002 (3)	0.021 (3)	−0.006 (3)
N3	0.027 (2)	0.043 (3)	0.028 (2)	0.000 (2)	0.0033 (19)	−0.003 (2)
N4	0.035 (3)	0.071 (4)	0.033 (3)	0.009 (3)	0.011 (2)	−0.012 (3)
S2	0.0323 (7)	0.0488 (10)	0.0352 (8)	0.0019 (7)	0.0081 (6)	0.0029 (7)
C23	0.033 (3)	0.040 (4)	0.035 (3)	−0.002 (3)	0.008 (3)	0.000 (3)
C24	0.032 (3)	0.047 (4)	0.040 (4)	−0.004 (3)	0.011 (3)	−0.008 (3)
C25	0.035 (3)	0.050 (4)	0.031 (3)	−0.002 (3)	0.011 (3)	−0.003 (3)
C26	0.036 (3)	0.042 (4)	0.032 (3)	0.000 (3)	0.004 (3)	−0.002 (3)
O1	0.036 (2)	0.072 (3)	0.031 (2)	−0.010 (2)	0.0048 (17)	−0.019 (2)
O2	0.037 (2)	0.108 (4)	0.033 (2)	−0.007 (2)	0.0091 (19)	−0.020 (2)
O3	0.037 (2)	0.071 (3)	0.033 (2)	−0.0045 (19)	0.0055 (17)	−0.017 (2)
O4	0.035 (2)	0.121 (4)	0.039 (2)	−0.010 (2)	0.013 (2)	−0.028 (2)

### Bis(2-amino-4,4,7,7-tetramethyl-4,5,6,7-tetrahydro-1,3-benzothiazol-3-ium) fumarate (IV) Geometric parameters (Å, º)

**Table:**

C1—C6	1.344 (7)	C12—C13	1.496 (7)
C1—C2	1.514 (7)	C12—S2	1.761 (5)
C1—S1	1.744 (5)	C13—C19	1.527 (8)
C2—C3A	1.520 (9)	C13—C14	1.532 (7)
C2—C8	1.524 (8)	C13—C20	1.533 (8)
C2—C9	1.551 (9)	C14—C15	1.526 (8)
C2—C3B	1.66 (3)	C14—H14A	0.9900
C3A—C4A	1.541 (14)	C14—H14B	0.9900
C3A—H3A1	0.9900	C15—C16	1.542 (7)
C3A—H3A2	0.9900	C15—H15A	0.9900
C4A—C5	1.575 (10)	C15—H15B	0.9900
C4A—H4A1	0.9900	C16—C17	1.513 (7)
C4A—H4A2	0.9900	C16—C22	1.523 (7)
C3B—C4B	1.53 (5)	C16—C21	1.536 (7)
C3B—H3B1	0.9900	C17—N3	1.404 (6)
C3B—H3B2	0.9900	C18—N4	1.319 (7)
C4B—C5	1.47 (2)	C18—N3	1.320 (6)
C4B—H4B1	0.9900	C18—S2	1.723 (5)
C4B—H4B2	0.9900	C19—H19A	0.9800
C5—C6	1.504 (7)	C19—H19B	0.9800
C5—C10	1.526 (8)	C19—H19C	0.9800
C5—C11	1.540 (8)	C20—H20A	0.9800
C6—N1	1.401 (6)	C20—H20B	0.9800
C7—N2	1.318 (6)	C20—H20C	0.9800
C7—N1	1.325 (6)	C21—H21A	0.9800
C7—S1	1.721 (5)	C21—H21B	0.9800
C8—H8A	0.9800	C21—H21C	0.9800
C8—H8B	0.9800	C22—H22A	0.9800
C8—H8C	0.9800	C22—H22B	0.9800
C9—H9A	0.9800	C22—H22C	0.9800
C9—H9B	0.9800	N3—H4N	0.96 (5)
C9—H9C	0.9800	N4—H5N	1.00 (6)
C10—H10A	0.9800	N4—H6N	0.81 (6)
C10—H10B	0.9800	C23—O1	1.251 (6)
C10—H10C	0.9800	C23—O2	1.258 (6)
C11—H11A	0.9800	C23—C24	1.484 (7)
C11—H11B	0.9800	C24—C25	1.321 (6)
C11—H11C	0.9800	C24—H24	0.9500
N1—H1N	0.85 (5)	C25—C26	1.483 (7)
N2—H2N	0.94 (6)	C25—H25	0.9500
N2—H3N	0.91 (6)	C26—O4	1.232 (6)
C12—C17	1.345 (7)	C26—O3	1.267 (6)
			
C6—C1—C2	126.7 (5)	C7—N2—H3N	119 (4)
C6—C1—S1	111.2 (4)	H2N—N2—H3N	118 (5)
C2—C1—S1	122.1 (4)	C7—S1—C1	90.3 (2)
C1—C2—C3A	106.1 (5)	C17—C12—C13	127.0 (5)
C1—C2—C8	109.5 (5)	C17—C12—S2	110.0 (4)
C3A—C2—C8	118.0 (6)	C13—C12—S2	122.9 (4)
C1—C2—C9	109.7 (5)	C12—C13—C19	110.8 (5)
C3A—C2—C9	105.5 (6)	C12—C13—C14	106.9 (4)
C8—C2—C9	107.7 (5)	C19—C13—C14	111.1 (5)
C1—C2—C3B	102.3 (11)	C12—C13—C20	110.9 (5)
C8—C2—C3B	89.8 (15)	C19—C13—C20	108.2 (5)
C9—C2—C3B	134.9 (15)	C14—C13—C20	108.8 (5)
C2—C3A—C4A	111.1 (9)	C15—C14—C13	113.0 (5)
C2—C3A—H3A1	109.4	C15—C14—H14A	109.0
C4A—C3A—H3A1	109.4	C13—C14—H14A	109.0
C2—C3A—H3A2	109.4	C15—C14—H14B	109.0
C4A—C3A—H3A2	109.4	C13—C14—H14B	109.0
H3A1—C3A—H3A2	108.0	H14A—C14—H14B	107.8
C3A—C4A—C5	111.7 (8)	C14—C15—C16	114.5 (5)
C3A—C4A—H4A1	109.3	C14—C15—H15A	108.6
C5—C4A—H4A1	109.3	C16—C15—H15A	108.6
C3A—C4A—H4A2	109.3	C14—C15—H15B	108.6
C5—C4A—H4A2	109.3	C16—C15—H15B	108.6
H4A1—C4A—H4A2	107.9	H15A—C15—H15B	107.6
C4B—C3B—C2	114 (3)	C17—C16—C22	109.7 (4)
C4B—C3B—H3B1	108.7	C17—C16—C21	110.2 (4)
C2—C3B—H3B1	108.7	C22—C16—C21	109.8 (5)
C4B—C3B—H3B2	108.7	C17—C16—C15	106.8 (4)
C2—C3B—H3B2	108.7	C22—C16—C15	111.4 (4)
H3B1—C3B—H3B2	107.6	C21—C16—C15	108.9 (5)
C5—C4B—C3B	112 (3)	C12—C17—N3	113.5 (4)
C5—C4B—H4B1	109.3	C12—C17—C16	125.6 (4)
C3B—C4B—H4B1	109.3	N3—C17—C16	120.9 (4)
C5—C4B—H4B2	109.3	N4—C18—N3	122.9 (5)
C3B—C4B—H4B2	109.3	N4—C18—S2	124.9 (4)
H4B1—C4B—H4B2	107.9	N3—C18—S2	112.1 (4)
C4B—C5—C6	112.1 (10)	C13—C19—H19A	109.5
C4B—C5—C10	121.4 (12)	C13—C19—H19B	109.5
C6—C5—C10	110.5 (5)	H19A—C19—H19B	109.5
C4B—C5—C11	92.1 (15)	C13—C19—H19C	109.5
C6—C5—C11	110.1 (4)	H19A—C19—H19C	109.5
C10—C5—C11	108.9 (5)	H19B—C19—H19C	109.5
C6—C5—C4A	105.1 (5)	C13—C20—H20A	109.5
C10—C5—C4A	106.4 (5)	C13—C20—H20B	109.5
C11—C5—C4A	115.8 (6)	H20A—C20—H20B	109.5
C1—C6—N1	112.5 (4)	C13—C20—H20C	109.5
C1—C6—C5	126.0 (4)	H20A—C20—H20C	109.5
N1—C6—C5	121.5 (4)	H20B—C20—H20C	109.5
N2—C7—N1	123.7 (5)	C16—C21—H21A	109.5
N2—C7—S1	124.4 (4)	C16—C21—H21B	109.5
N1—C7—S1	111.8 (4)	H21A—C21—H21B	109.5
C2—C8—H8A	109.5	C16—C21—H21C	109.5
C2—C8—H8B	109.5	H21A—C21—H21C	109.5
H8A—C8—H8B	109.5	H21B—C21—H21C	109.5
C2—C8—H8C	109.5	C16—C22—H22A	109.5
H8A—C8—H8C	109.5	C16—C22—H22B	109.5
H8B—C8—H8C	109.5	H22A—C22—H22B	109.5
C2—C9—H9A	109.5	C16—C22—H22C	109.5
C2—C9—H9B	109.5	H22A—C22—H22C	109.5
H9A—C9—H9B	109.5	H22B—C22—H22C	109.5
C2—C9—H9C	109.5	C18—N3—C17	113.9 (4)
H9A—C9—H9C	109.5	C18—N3—H4N	123 (3)
H9B—C9—H9C	109.5	C17—N3—H4N	123 (3)
C5—C10—H10A	109.5	C18—N4—H5N	112 (3)
C5—C10—H10B	109.5	C18—N4—H6N	115 (4)
H10A—C10—H10B	109.5	H5N—N4—H6N	130 (5)
C5—C10—H10C	109.5	C18—S2—C12	90.5 (2)
H10A—C10—H10C	109.5	O1—C23—O2	124.2 (5)
H10B—C10—H10C	109.5	O1—C23—C24	118.1 (5)
C5—C11—H11A	109.5	O2—C23—C24	117.7 (5)
C5—C11—H11B	109.5	C25—C24—C23	124.6 (5)
H11A—C11—H11B	109.5	C25—C24—H24	117.7
C5—C11—H11C	109.5	C23—C24—H24	117.7
H11A—C11—H11C	109.5	C24—C25—C26	124.6 (5)
H11B—C11—H11C	109.5	C24—C25—H25	117.7
C7—N1—C6	114.1 (4)	C26—C25—H25	117.7
C7—N1—H1N	119 (3)	O4—C26—O3	124.4 (5)
C6—N1—H1N	126 (3)	O4—C26—C25	119.2 (5)
C7—N2—H2N	117 (4)	O3—C26—C25	116.3 (5)
			
C6—C1—C2—C3A	−15.4 (9)	N1—C7—S1—C1	−0.6 (4)
S1—C1—C2—C3A	165.0 (6)	C6—C1—S1—C7	0.8 (4)
C6—C1—C2—C8	113.1 (6)	C2—C1—S1—C7	−179.5 (5)
S1—C1—C2—C8	−66.6 (6)	C17—C12—C13—C19	107.5 (6)
C6—C1—C2—C9	−128.9 (6)	S2—C12—C13—C19	−75.3 (6)
S1—C1—C2—C9	51.4 (6)	C17—C12—C13—C14	−13.7 (7)
C6—C1—C2—C3B	18.8 (17)	S2—C12—C13—C14	163.5 (4)
S1—C1—C2—C3B	−160.8 (16)	C17—C12—C13—C20	−132.2 (6)
C1—C2—C3A—C4A	47.8 (10)	S2—C12—C13—C20	45.0 (6)
C8—C2—C3A—C4A	−75.5 (9)	C12—C13—C14—C15	44.1 (6)
C9—C2—C3A—C4A	164.1 (8)	C19—C13—C14—C15	−77.0 (6)
C2—C3A—C4A—C5	−69.2 (12)	C20—C13—C14—C15	163.9 (5)
C1—C2—C3B—C4B	−48 (3)	C13—C14—C15—C16	−62.5 (7)
C8—C2—C3B—C4B	−158 (3)	C14—C15—C16—C17	40.9 (6)
C9—C2—C3B—C4B	87 (3)	C14—C15—C16—C22	−78.9 (6)
C2—C3B—C4B—C5	62 (4)	C14—C15—C16—C21	159.9 (5)
C3B—C4B—C5—C6	−38 (3)	C13—C12—C17—N3	176.7 (5)
C3B—C4B—C5—C10	95 (3)	S2—C12—C17—N3	−0.8 (6)
C3B—C4B—C5—C11	−151 (3)	C13—C12—C17—C16	−3.4 (8)
C3A—C4A—C5—C6	47.3 (10)	S2—C12—C17—C16	179.1 (4)
C3A—C4A—C5—C10	164.5 (8)	C22—C16—C17—C12	111.1 (6)
C3A—C4A—C5—C11	−74.4 (10)	C21—C16—C17—C12	−127.9 (5)
C2—C1—C6—N1	179.6 (5)	C15—C16—C17—C12	−9.8 (7)
S1—C1—C6—N1	−0.7 (5)	C22—C16—C17—N3	−69.1 (6)
C2—C1—C6—C5	−0.9 (9)	C21—C16—C17—N3	51.9 (6)
S1—C1—C6—C5	178.8 (4)	C15—C16—C17—N3	170.1 (5)
C4B—C5—C6—C1	9.7 (17)	N4—C18—N3—C17	−178.6 (5)
C10—C5—C6—C1	−129.1 (5)	S2—C18—N3—C17	−1.4 (6)
C11—C5—C6—C1	110.6 (6)	C12—C17—N3—C18	1.5 (6)
C4A—C5—C6—C1	−14.7 (8)	C16—C17—N3—C18	−178.4 (4)
C4B—C5—C6—N1	−170.9 (16)	N4—C18—S2—C12	177.9 (5)
C10—C5—C6—N1	50.4 (6)	N3—C18—S2—C12	0.8 (4)
C11—C5—C6—N1	−69.9 (6)	C17—C12—S2—C18	0.0 (4)
C4A—C5—C6—N1	164.7 (6)	C13—C12—S2—C18	−177.6 (4)
N2—C7—N1—C6	177.7 (5)	O1—C23—C24—C25	177.5 (5)
S1—C7—N1—C6	0.3 (6)	O2—C23—C24—C25	−2.4 (8)
C1—C6—N1—C7	0.3 (6)	C23—C24—C25—C26	177.5 (5)
C5—C6—N1—C7	−179.2 (5)	C24—C25—C26—O4	−0.8 (8)
N2—C7—S1—C1	−177.9 (5)	C24—C25—C26—O3	179.9 (5)

### Bis(2-amino-4,4,7,7-tetramethyl-4,5,6,7-tetrahydro-1,3-benzothiazol-3-ium) fumarate (IV) Hydrogen-bond geometry (Å, º)

**Table:**

*D*—H···*A*	*D*—H	H···*A*	*D*···*A*	*D*—H···*A*
N1—H1*N*···O1	0.85 (5)	1.84 (5)	2.670 (5)	167 (5)
N2—H2*N*···O2	0.94 (6)	1.77 (6)	2.704 (6)	177 (5)
N2—H3*N*···O4^i^	0.91 (6)	1.90 (6)	2.746 (6)	153 (5)
N3—H4*N*···O3	0.96 (5)	1.67 (5)	2.617 (5)	169 (5)
N4—H5*N*···O4	1.00 (6)	1.75 (6)	2.754 (7)	178 (5)
N4—H6*N*···O2^ii^	0.81 (6)	2.09 (6)	2.763 (6)	141 (6)

Symmetry codes: (i) *x*+1, *y*, *z*; (ii) *x*−1, *y*, *z*.

### Bis(2-amino-4,4,7,7-tetramethyl-4,5,6,7-tetrahydro-1,3-benzothiazol-3-ium) succinate–2-amino-4,4,7,7-tetramethyl-4,5,6,7-tetrahydro-1,3-benzothiazol-3-ium hydrogen succinate 4,4,7,7-tetramethyl-3a,5,6,7a-tetrahydrobenzothiazol-2-ylamine (1/1) (V) Crystal data

**Table:**

1.5C_11_H_19_N_2_S^+^·0.5C_4_H_4_O_4_^2^^−^·0.5C_4_H_5_O_4_^−^·0.5C_11_H_18_N_2_S	*F*(000) = 580
*M**_r_* = 538.75	*D*_x_ = 1.226 Mg m^−^^3^
Monoclinic, *P*2_1_	Mo *K*α radiation, λ = 0.71073 Å
*a* = 8.9437 (3) Å	Cell parameters from 9613 reflections
*b* = 14.7253 (4) Å	θ = 2.3–28.0°
*c* = 11.2676 (4) Å	µ = 0.22 mm^−^^1^
β = 100.493 (3)°	*T* = 173 K
*V* = 1459.11 (8) Å^3^	Plate, colourless
*Z* = 2	0.32 × 0.15 × 0.02 mm

### Bis(2-amino-4,4,7,7-tetramethyl-4,5,6,7-tetrahydro-1,3-benzothiazol-3-ium) succinate–2-amino-4,4,7,7-tetramethyl-4,5,6,7-tetrahydro-1,3-benzothiazol-3-ium hydrogen succinate 4,4,7,7-tetramethyl-3a,5,6,7a-tetrahydrobenzothiazol-2-ylamine (1/1) (V) Data collection

**Table:**

Rigaku XtaLAB P200 HPC diffractometer	6272 independent reflections
Radiation source: rotating anode, Rigaku FR-X	5511 reflections with *I* > 2σ(*I*)
Rigaku Osmic Confocal Optical System monochromator	*R*_int_ = 0.034
ω scans	θ_max_ = 29.1°, θ_min_ = 2.3°
Absorption correction: multi-scan (CrysAlis PRO; Rigaku, 2017)	*h* = −10→12
*T*_min_ = 0.779, *T*_max_ = 1.000	*k* = −19→19
19079 measured reflections	*l* = −14→14

### Bis(2-amino-4,4,7,7-tetramethyl-4,5,6,7-tetrahydro-1,3-benzothiazol-3-ium) succinate–2-amino-4,4,7,7-tetramethyl-4,5,6,7-tetrahydro-1,3-benzothiazol-3-ium hydrogen succinate 4,4,7,7-tetramethyl-3a,5,6,7a-tetrahydrobenzothiazol-2-ylamine (1/1) (V) Refinement

**Table:**

Refinement on *F*^2^	Hydrogen site location: mixed
Least-squares matrix: full	H atoms treated by a mixture of independent and constrained refinement
*R*[*F*^2^ > 2σ(*F*^2^)] = 0.039	*w* = 1/[σ^2^(*F*_o_^2^) + (0.0529*P*)^2^ + 0.1056*P*] where *P* = (*F*_o_^2^ + 2*F*_c_^2^)/3
*wR*(*F*^2^) = 0.094	(Δ/σ)_max_ = 0.001
*S* = 1.04	Δρ_max_ = 0.26 e Å^−^^3^
6272 reflections	Δρ_min_ = −0.25 e Å^−^^3^
354 parameters	Absolute structure: Flack *x* determined using 2211 quotients [(*I*^+^)-(*I*^-^)]/[(*I*^+^)+(*I*^-^)] (Parsons *et al.*, 2013)
1 restraint	Absolute structure parameter: 0.00 (3)
Primary atom site location: structure-invariant direct methods	

### Bis(2-amino-4,4,7,7-tetramethyl-4,5,6,7-tetrahydro-1,3-benzothiazol-3-ium) succinate–2-amino-4,4,7,7-tetramethyl-4,5,6,7-tetrahydro-1,3-benzothiazol-3-ium hydrogen succinate 4,4,7,7-tetramethyl-3a,5,6,7a-tetrahydrobenzothiazol-2-ylamine (1/1) (V) Special details

**Table:**

Geometry. All esds (except the esd in the dihedral angle between two l.s. planes) are estimated using the full covariance matrix. The cell esds are taken into account individually in the estimation of esds in distances, angles and torsion angles; correlations between esds in cell parameters are only used when they are defined by crystal symmetry. An approximate (isotropic) treatment of cell esds is used for estimating esds involving l.s. planes.

### Bis(2-amino-4,4,7,7-tetramethyl-4,5,6,7-tetrahydro-1,3-benzothiazol-3-ium) succinate–2-amino-4,4,7,7-tetramethyl-4,5,6,7-tetrahydro-1,3-benzothiazol-3-ium hydrogen succinate 4,4,7,7-tetramethyl-3a,5,6,7a-tetrahydrobenzothiazol-2-ylamine (1/1) (V) Fractional atomic coordinates and isotropic or equivalent isotropic displacement parameters (Å^2^)

**Table:**

	*x*	*y*	*z*	*U*_iso_*/*U*_eq_	Occ. (<1)
C1	1.2888 (3)	0.39542 (19)	0.6860 (2)	0.0263 (6)	
C2	1.3946 (3)	0.4407 (2)	0.7881 (3)	0.0357 (7)	
C3A	1.2962 (6)	0.5069 (4)	0.8461 (5)	0.0365 (17)*	0.593 (11)
H3A1	1.2689	0.5598	0.7923	0.044*	0.593 (11)
H3A2	1.3548	0.5293	0.9237	0.044*	0.593 (11)
C4A	1.1500 (6)	0.4594 (5)	0.8685 (6)	0.0366 (16)*	0.593 (11)
H4A1	1.0979	0.4985	0.9198	0.044*	0.593 (11)
H4A2	1.1768	0.4015	0.9118	0.044*	0.593 (11)
C3B	1.2917 (10)	0.4598 (8)	0.8865 (9)	0.049 (3)*	0.407 (11)
H3B1	1.2754	0.4020	0.9271	0.059*	0.407 (11)
H3B2	1.3468	0.5015	0.9483	0.059*	0.407 (11)
C4B	1.1399 (9)	0.5005 (7)	0.8353 (8)	0.038 (2)*	0.407 (11)
H4B1	1.1564	0.5586	0.7954	0.046*	0.407 (11)
H4B2	1.0860	0.5143	0.9025	0.046*	0.407 (11)
C5	1.0393 (3)	0.4400 (2)	0.7449 (3)	0.0308 (6)	
C6	1.1354 (3)	0.39467 (18)	0.6659 (2)	0.0236 (6)	
C7	1.1723 (3)	0.30960 (19)	0.5050 (2)	0.0265 (6)	
C8	1.4933 (5)	0.3708 (3)	0.8639 (4)	0.0612 (11)	
H8A	1.5574	0.4010	0.9324	0.092*	
H8B	1.5579	0.3404	0.8146	0.092*	
H8C	1.4285	0.3258	0.8939	0.092*	
C9	1.5000 (5)	0.5061 (3)	0.7370 (4)	0.0690 (12)	
H9A	1.5592	0.5414	0.8030	0.103*	
H9B	1.4390	0.5474	0.6793	0.103*	
H9C	1.5691	0.4714	0.6961	0.103*	
C10	0.9296 (4)	0.3719 (2)	0.7834 (3)	0.0480 (9)	
H10A	0.8715	0.4014	0.8385	0.072*	
H10B	0.9869	0.3207	0.8245	0.072*	
H10C	0.8596	0.3499	0.7121	0.072*	
C11	0.9468 (5)	0.5170 (3)	0.6769 (4)	0.0669 (13)	
H11A	0.8820	0.5444	0.7285	0.100*	
H11B	0.8830	0.4928	0.6036	0.100*	
H11C	1.0158	0.5631	0.6547	0.100*	
N1	1.0710 (3)	0.34631 (16)	0.5629 (2)	0.0246 (5)	
H1N	0.968 (4)	0.335 (2)	0.534 (3)	0.030*	
N2	1.1368 (3)	0.2592 (2)	0.4072 (2)	0.0397 (7)	
H2N	1.209 (4)	0.241 (3)	0.369 (3)	0.048*	
H3N	1.047 (4)	0.257 (3)	0.374 (3)	0.048*	
S1	1.35670 (7)	0.33342 (5)	0.57387 (6)	0.03090 (18)	
C12	−0.1518 (3)	0.11232 (19)	−0.1824 (3)	0.0261 (6)	
C13	−0.2459 (4)	0.0700 (2)	−0.2927 (3)	0.0345 (7)	
C14	−0.1355 (4)	0.0472 (2)	−0.3784 (3)	0.0405 (8)	
H14A	−0.1871	0.0066	−0.4431	0.049*	
H14B	−0.1081	0.1038	−0.4167	0.049*	
C15	0.0097 (4)	0.0011 (2)	−0.3133 (3)	0.0426 (8)	
H15A	0.0715	−0.0172	−0.3738	0.051*	
H15B	−0.0183	−0.0547	−0.2736	0.051*	
C16	0.1071 (3)	0.06150 (19)	−0.2180 (3)	0.0295 (6)	
C17	0.0007 (3)	0.11035 (18)	−0.1495 (2)	0.0245 (6)	
C18	−0.0513 (3)	0.19535 (18)	0.0048 (2)	0.0263 (6)	
C19	−0.3270 (5)	−0.0149 (3)	−0.2585 (4)	0.0581 (10)	
H19A	−0.3866	−0.0421	−0.3315	0.087*	
H19B	−0.3950	0.0018	−0.2030	0.087*	
H19C	−0.2516	−0.0588	−0.2193	0.087*	
C20	−0.3652 (4)	0.1381 (2)	−0.3552 (3)	0.0459 (8)	
H20A	−0.4216	0.1112	−0.4295	0.069*	
H20B	−0.3141	0.1936	−0.3748	0.069*	
H20C	−0.4358	0.1530	−0.3010	0.069*	
C21	0.2173 (4)	0.0007 (2)	−0.1322 (3)	0.0446 (8)	
H21A	0.2785	−0.0351	−0.1789	0.067*	
H21B	0.1593	−0.0401	−0.0890	0.067*	
H21C	0.2844	0.0388	−0.0739	0.067*	
C22	0.1982 (4)	0.1307 (2)	−0.2773 (3)	0.0426 (8)	
H22A	0.2681	0.0986	−0.3203	0.064*	
H22B	0.2564	0.1697	−0.2150	0.064*	
H22C	0.1283	0.1680	−0.3344	0.064*	
N3	0.0575 (3)	0.15834 (16)	−0.0442 (2)	0.0248 (5)	
H4N	0.153 (9)	0.165 (5)	−0.011 (6)	0.030*	0.50 (5)
N4	−0.0255 (3)	0.2469 (2)	0.1041 (2)	0.0387 (7)	
H5N	−0.096 (4)	0.249 (3)	0.142 (3)	0.046*	
H6N	0.073 (4)	0.245 (2)	0.147 (3)	0.046*	
S2	−0.23122 (8)	0.17442 (5)	−0.07649 (6)	0.02918 (18)	
C26	0.3743 (3)	0.22426 (19)	0.1462 (3)	0.0271 (6)	
C25	0.5352 (3)	0.2536 (2)	0.1889 (3)	0.0298 (6)	
H25A	0.5538	0.3096	0.1451	0.036*	
H25B	0.6039	0.2060	0.1676	0.036*	
C24	0.5760 (3)	0.2717 (2)	0.3238 (3)	0.0300 (7)	
H24A	0.5202	0.3264	0.3427	0.036*	
H24B	0.5406	0.2199	0.3672	0.036*	
C23	0.7441 (3)	0.2859 (2)	0.3709 (3)	0.0284 (6)	
O1	0.7810 (2)	0.32184 (16)	0.47272 (18)	0.0379 (5)	
O2	0.8363 (2)	0.2585 (2)	0.3079 (2)	0.0575 (7)	
O3	0.3395 (3)	0.20074 (16)	0.03486 (19)	0.0388 (6)	
H1O	0.249 (11)	0.191 (5)	0.008 (7)	0.047*	0.50 (5)
O4	0.2832 (2)	0.2213 (2)	0.21545 (19)	0.0500 (7)	

### Bis(2-amino-4,4,7,7-tetramethyl-4,5,6,7-tetrahydro-1,3-benzothiazol-3-ium) succinate–2-amino-4,4,7,7-tetramethyl-4,5,6,7-tetrahydro-1,3-benzothiazol-3-ium hydrogen succinate 4,4,7,7-tetramethyl-3a,5,6,7a-tetrahydrobenzothiazol-2-ylamine (1/1) (V) Atomic displacement parameters (Å^2^)

**Table:**

	*U*^11^	*U*^22^	*U*^33^	*U*^12^	*U*^13^	*U*^23^
C1	0.0280 (14)	0.0258 (13)	0.0253 (14)	0.0017 (11)	0.0051 (11)	−0.0003 (12)
C2	0.0308 (15)	0.0380 (16)	0.0364 (17)	0.0002 (13)	0.0009 (13)	−0.0095 (15)
C5	0.0322 (15)	0.0316 (15)	0.0295 (15)	−0.0011 (13)	0.0082 (13)	−0.0073 (13)
C6	0.0287 (14)	0.0218 (12)	0.0207 (13)	−0.0020 (11)	0.0050 (11)	0.0005 (11)
C7	0.0236 (14)	0.0331 (15)	0.0223 (14)	0.0018 (11)	0.0032 (11)	0.0001 (12)
C8	0.063 (2)	0.059 (2)	0.048 (2)	−0.019 (2)	−0.0263 (19)	0.0101 (19)
C9	0.069 (3)	0.047 (2)	0.079 (3)	−0.026 (2)	−0.018 (2)	0.003 (2)
C10	0.054 (2)	0.0462 (19)	0.053 (2)	0.0011 (17)	0.0319 (18)	0.0051 (17)
C11	0.079 (3)	0.048 (2)	0.088 (3)	0.031 (2)	0.052 (3)	0.023 (2)
N1	0.0226 (12)	0.0286 (12)	0.0226 (12)	−0.0009 (10)	0.0037 (9)	−0.0015 (10)
N2	0.0287 (14)	0.0613 (19)	0.0288 (15)	0.0056 (13)	0.0044 (12)	−0.0156 (14)
S1	0.0231 (3)	0.0400 (4)	0.0290 (4)	0.0043 (3)	0.0032 (3)	−0.0045 (3)
C12	0.0266 (14)	0.0253 (13)	0.0267 (14)	−0.0014 (11)	0.0058 (12)	0.0007 (12)
C13	0.0337 (16)	0.0343 (15)	0.0331 (16)	−0.0093 (13)	−0.0005 (13)	−0.0047 (14)
C14	0.0425 (18)	0.0461 (18)	0.0305 (16)	−0.0013 (14)	0.0000 (14)	−0.0137 (15)
C15	0.0437 (18)	0.0420 (17)	0.0408 (18)	0.0018 (15)	0.0044 (15)	−0.0170 (15)
C16	0.0303 (14)	0.0295 (14)	0.0287 (15)	0.0027 (12)	0.0052 (12)	−0.0028 (13)
C17	0.0276 (14)	0.0231 (12)	0.0225 (14)	−0.0007 (11)	0.0038 (11)	0.0016 (11)
C18	0.0262 (14)	0.0297 (15)	0.0225 (14)	0.0023 (11)	0.0032 (12)	0.0017 (11)
C19	0.062 (2)	0.048 (2)	0.061 (2)	−0.0282 (18)	0.004 (2)	−0.0068 (19)
C20	0.0333 (17)	0.060 (2)	0.0387 (19)	0.0004 (16)	−0.0078 (14)	−0.0049 (17)
C21	0.0418 (18)	0.0429 (18)	0.0472 (19)	0.0173 (15)	0.0028 (16)	−0.0068 (16)
C22	0.0396 (18)	0.0499 (19)	0.0433 (19)	−0.0023 (15)	0.0206 (16)	−0.0042 (16)
N3	0.0218 (11)	0.0306 (13)	0.0210 (12)	0.0003 (10)	0.0015 (9)	−0.0004 (10)
N4	0.0286 (14)	0.0594 (17)	0.0277 (15)	0.0047 (13)	0.0038 (12)	−0.0121 (13)
S2	0.0230 (3)	0.0361 (4)	0.0287 (4)	0.0008 (3)	0.0053 (3)	0.0016 (3)
C26	0.0268 (14)	0.0289 (14)	0.0248 (14)	0.0020 (12)	0.0021 (12)	−0.0022 (12)
C25	0.0281 (15)	0.0363 (15)	0.0243 (15)	−0.0015 (12)	0.0031 (12)	−0.0019 (13)
C24	0.0250 (14)	0.0386 (16)	0.0256 (15)	0.0016 (12)	0.0025 (12)	−0.0064 (13)
C23	0.0279 (15)	0.0321 (14)	0.0251 (15)	−0.0012 (12)	0.0045 (12)	−0.0019 (13)
O1	0.0275 (10)	0.0581 (14)	0.0274 (11)	−0.0050 (10)	0.0029 (8)	−0.0178 (11)
O2	0.0259 (12)	0.109 (2)	0.0368 (13)	−0.0003 (13)	0.0035 (10)	−0.0313 (14)
O3	0.0263 (11)	0.0618 (16)	0.0273 (12)	−0.0077 (10)	0.0022 (9)	−0.0105 (10)
O4	0.0280 (11)	0.0919 (19)	0.0312 (12)	−0.0076 (12)	0.0084 (10)	−0.0186 (13)

### Bis(2-amino-4,4,7,7-tetramethyl-4,5,6,7-tetrahydro-1,3-benzothiazol-3-ium) succinate–2-amino-4,4,7,7-tetramethyl-4,5,6,7-tetrahydro-1,3-benzothiazol-3-ium hydrogen succinate 4,4,7,7-tetramethyl-3a,5,6,7a-tetrahydrobenzothiazol-2-ylamine (1/1) (V) Geometric parameters (Å, º)

**Table:**

C1—C6	1.349 (4)	C13—C19	1.530 (4)
C1—C2	1.506 (4)	C13—C20	1.538 (5)
C1—S1	1.754 (3)	C13—C14	1.538 (4)
C2—C8	1.515 (5)	C14—C15	1.528 (5)
C2—C9	1.531 (5)	C14—H14A	0.9900
C2—C3A	1.537 (6)	C14—H14B	0.9900
C2—C3B	1.589 (10)	C15—C16	1.536 (4)
C3A—C4A	1.544 (9)	C15—H15A	0.9900
C3A—H3A1	0.9900	C15—H15B	0.9900
C3A—H3A2	0.9900	C16—C17	1.512 (4)
C4A—C5	1.581 (6)	C16—C22	1.532 (4)
C4A—H4A1	0.9900	C16—C21	1.536 (4)
C4A—H4A2	0.9900	C17—N3	1.395 (4)
C3B—C4B	1.499 (14)	C18—N3	1.321 (3)
C3B—H3B1	0.9900	C18—N4	1.337 (4)
C3B—H3B2	0.9900	C18—S2	1.728 (3)
C4B—C5	1.519 (8)	C19—H19A	0.9800
C4B—H4B1	0.9900	C19—H19B	0.9800
C4B—H4B2	0.9900	C19—H19C	0.9800
C5—C6	1.502 (4)	C20—H20A	0.9800
C5—C10	1.520 (4)	C20—H20B	0.9800
C5—C11	1.525 (5)	C20—H20C	0.9800
C6—N1	1.394 (3)	C21—H21A	0.9800
C7—N2	1.319 (4)	C21—H21B	0.9800
C7—N1	1.325 (3)	C21—H21C	0.9800
C7—S1	1.726 (3)	C22—H22A	0.9800
C8—H8A	0.9800	C22—H22B	0.9800
C8—H8B	0.9800	C22—H22C	0.9800
C8—H8C	0.9800	N3—H4N	0.87 (8)
C9—H9A	0.9800	N4—H5N	0.82 (4)
C9—H9B	0.9800	N4—H6N	0.92 (4)
C9—H9C	0.9800	C26—O4	1.228 (3)
C10—H10A	0.9800	C26—O3	1.283 (4)
C10—H10B	0.9800	C26—C25	1.496 (4)
C10—H10C	0.9800	C25—C24	1.521 (4)
C11—H11A	0.9800	C25—H25A	0.9900
C11—H11B	0.9800	C25—H25B	0.9900
C11—H11C	0.9800	C24—C23	1.515 (4)
N1—H1N	0.93 (3)	C24—H24A	0.9900
N2—H2N	0.89 (4)	C24—H24B	0.9900
N2—H3N	0.82 (4)	C23—O2	1.249 (4)
C12—C17	1.348 (4)	C23—O1	1.252 (3)
C12—C13	1.503 (4)	O3—H1O	0.82 (10)
C12—S2	1.753 (3)		
			
C6—C1—C2	127.6 (3)	C17—C12—S2	109.8 (2)
C6—C1—S1	110.5 (2)	C13—C12—S2	123.0 (2)
C2—C1—S1	122.0 (2)	C12—C13—C19	110.5 (3)
C1—C2—C8	110.5 (3)	C12—C13—C20	110.2 (3)
C1—C2—C9	109.6 (3)	C19—C13—C20	109.1 (3)
C8—C2—C9	107.7 (3)	C12—C13—C14	106.4 (2)
C1—C2—C3A	106.1 (3)	C19—C13—C14	111.6 (3)
C8—C2—C3A	120.8 (4)	C20—C13—C14	109.1 (3)
C9—C2—C3A	101.6 (4)	C15—C14—C13	112.5 (3)
C1—C2—C3B	104.4 (4)	C15—C14—H14A	109.1
C8—C2—C3B	94.5 (5)	C13—C14—H14A	109.1
C9—C2—C3B	128.7 (5)	C15—C14—H14B	109.1
C2—C3A—C4A	110.5 (5)	C13—C14—H14B	109.1
C2—C3A—H3A1	109.6	H14A—C14—H14B	107.8
C4A—C3A—H3A1	109.6	C14—C15—C16	113.7 (3)
C2—C3A—H3A2	109.6	C14—C15—H15A	108.8
C4A—C3A—H3A2	109.6	C16—C15—H15A	108.8
H3A1—C3A—H3A2	108.1	C14—C15—H15B	108.8
C3A—C4A—C5	110.5 (5)	C16—C15—H15B	108.8
C3A—C4A—H4A1	109.5	H15A—C15—H15B	107.7
C5—C4A—H4A1	109.5	C17—C16—C22	109.9 (2)
C3A—C4A—H4A2	109.5	C17—C16—C21	110.3 (2)
C5—C4A—H4A2	109.5	C22—C16—C21	109.3 (3)
H4A1—C4A—H4A2	108.1	C17—C16—C15	107.6 (2)
C4B—C3B—C2	113.6 (8)	C22—C16—C15	111.0 (3)
C4B—C3B—H3B1	108.8	C21—C16—C15	108.7 (3)
C2—C3B—H3B1	108.8	C12—C17—N3	114.6 (2)
C4B—C3B—H3B2	108.8	C12—C17—C16	124.7 (2)
C2—C3B—H3B2	108.8	N3—C17—C16	120.7 (2)
H3B1—C3B—H3B2	107.7	N3—C18—N4	123.8 (3)
C3B—C4B—C5	114.1 (8)	N3—C18—S2	113.0 (2)
C3B—C4B—H4B1	108.7	N4—C18—S2	123.2 (2)
C5—C4B—H4B1	108.7	C13—C19—H19A	109.5
C3B—C4B—H4B2	108.7	C13—C19—H19B	109.5
C5—C4B—H4B2	108.7	H19A—C19—H19B	109.5
H4B1—C4B—H4B2	107.6	C13—C19—H19C	109.5
C6—C5—C4B	109.0 (4)	H19A—C19—H19C	109.5
C6—C5—C10	110.2 (2)	H19B—C19—H19C	109.5
C4B—C5—C10	122.0 (4)	C13—C20—H20A	109.5
C6—C5—C11	110.4 (3)	C13—C20—H20B	109.5
C4B—C5—C11	95.9 (5)	H20A—C20—H20B	109.5
C10—C5—C11	108.3 (3)	C13—C20—H20C	109.5
C6—C5—C4A	105.6 (3)	H20A—C20—H20C	109.5
C10—C5—C4A	101.6 (3)	H20B—C20—H20C	109.5
C11—C5—C4A	120.2 (4)	C16—C21—H21A	109.5
C1—C6—N1	113.4 (2)	C16—C21—H21B	109.5
C1—C6—C5	124.8 (3)	H21A—C21—H21B	109.5
N1—C6—C5	121.7 (2)	C16—C21—H21C	109.5
N2—C7—N1	124.0 (3)	H21A—C21—H21C	109.5
N2—C7—S1	123.7 (2)	H21B—C21—H21C	109.5
N1—C7—S1	112.3 (2)	C16—C22—H22A	109.5
C2—C8—H8A	109.5	C16—C22—H22B	109.5
C2—C8—H8B	109.5	H22A—C22—H22B	109.5
H8A—C8—H8B	109.5	C16—C22—H22C	109.5
C2—C8—H8C	109.5	H22A—C22—H22C	109.5
H8A—C8—H8C	109.5	H22B—C22—H22C	109.5
H8B—C8—H8C	109.5	C18—N3—C17	112.5 (2)
C2—C9—H9A	109.5	C18—N3—H4N	121 (4)
C2—C9—H9B	109.5	C17—N3—H4N	127 (4)
H9A—C9—H9B	109.5	C18—N4—H5N	115 (3)
C2—C9—H9C	109.5	C18—N4—H6N	115 (2)
H9A—C9—H9C	109.5	H5N—N4—H6N	118 (3)
H9B—C9—H9C	109.5	C18—S2—C12	90.03 (13)
C5—C10—H10A	109.5	O4—C26—O3	122.7 (3)
C5—C10—H10B	109.5	O4—C26—C25	121.1 (3)
H10A—C10—H10B	109.5	O3—C26—C25	116.1 (2)
C5—C10—H10C	109.5	C26—C25—C24	114.4 (2)
H10A—C10—H10C	109.5	C26—C25—H25A	108.7
H10B—C10—H10C	109.5	C24—C25—H25A	108.7
C5—C11—H11A	109.5	C26—C25—H25B	108.7
C5—C11—H11B	109.5	C24—C25—H25B	108.7
H11A—C11—H11B	109.5	H25A—C25—H25B	107.6
C5—C11—H11C	109.5	C23—C24—C25	114.6 (2)
H11A—C11—H11C	109.5	C23—C24—H24A	108.6
H11B—C11—H11C	109.5	C25—C24—H24A	108.6
C7—N1—C6	113.7 (2)	C23—C24—H24B	108.6
C7—N1—H1N	118.6 (18)	C25—C24—H24B	108.6
C6—N1—H1N	127.6 (18)	H24A—C24—H24B	107.6
C7—N2—H2N	120 (2)	O2—C23—O1	124.5 (3)
C7—N2—H3N	117 (3)	O2—C23—C24	118.2 (2)
H2N—N2—H3N	121 (4)	O1—C23—C24	117.3 (2)
C7—S1—C1	90.11 (13)	C26—O3—H1O	117 (5)
C17—C12—C13	127.2 (3)		
			
C6—C1—C2—C8	117.8 (4)	N1—C7—S1—C1	0.3 (2)
S1—C1—C2—C8	−61.8 (3)	C6—C1—S1—C7	0.1 (2)
C6—C1—C2—C9	−123.7 (3)	C2—C1—S1—C7	179.7 (2)
S1—C1—C2—C9	56.7 (3)	C17—C12—C13—C19	105.9 (4)
C6—C1—C2—C3A	−14.8 (5)	S2—C12—C13—C19	−74.9 (3)
S1—C1—C2—C3A	165.7 (3)	C17—C12—C13—C20	−133.5 (3)
C6—C1—C2—C3B	17.2 (6)	S2—C12—C13—C20	45.7 (3)
S1—C1—C2—C3B	−162.3 (5)	C17—C12—C13—C14	−15.3 (4)
C1—C2—C3A—C4A	47.7 (6)	S2—C12—C13—C14	163.9 (2)
C8—C2—C3A—C4A	−78.9 (5)	C12—C13—C14—C15	45.6 (3)
C9—C2—C3A—C4A	162.2 (5)	C19—C13—C14—C15	−74.9 (3)
C2—C3A—C4A—C5	−70.2 (6)	C20—C13—C14—C15	164.5 (3)
C1—C2—C3B—C4B	−46.1 (9)	C13—C14—C15—C16	−63.9 (4)
C8—C2—C3B—C4B	−158.7 (8)	C14—C15—C16—C17	42.2 (3)
C9—C2—C3B—C4B	84.4 (9)	C14—C15—C16—C22	−78.1 (3)
C2—C3B—C4B—C5	62.4 (12)	C14—C15—C16—C21	161.6 (3)
C3B—C4B—C5—C6	−40.4 (10)	C13—C12—C17—N3	177.9 (3)
C3B—C4B—C5—C10	89.8 (8)	S2—C12—C17—N3	−1.4 (3)
C3B—C4B—C5—C11	−154.4 (8)	C13—C12—C17—C16	−1.9 (4)
C3A—C4A—C5—C6	49.7 (6)	S2—C12—C17—C16	178.8 (2)
C3A—C4A—C5—C10	164.7 (5)	C22—C16—C17—C12	110.0 (3)
C3A—C4A—C5—C11	−75.9 (5)	C21—C16—C17—C12	−129.4 (3)
C2—C1—C6—N1	−179.9 (3)	C15—C16—C17—C12	−11.0 (4)
S1—C1—C6—N1	−0.4 (3)	C22—C16—C17—N3	−69.8 (3)
C2—C1—C6—C5	−0.4 (5)	C21—C16—C17—N3	50.8 (3)
S1—C1—C6—C5	179.2 (2)	C15—C16—C17—N3	169.2 (3)
C4B—C5—C6—C1	10.6 (6)	N4—C18—N3—C17	−177.9 (3)
C10—C5—C6—C1	−125.7 (3)	S2—C18—N3—C17	−0.6 (3)
C11—C5—C6—C1	114.7 (3)	C12—C17—N3—C18	1.3 (3)
C4A—C5—C6—C1	−16.8 (4)	C16—C17—N3—C18	−178.9 (2)
C4B—C5—C6—N1	−169.9 (5)	N3—C18—S2—C12	−0.2 (2)
C10—C5—C6—N1	53.8 (4)	N4—C18—S2—C12	177.2 (3)
C11—C5—C6—N1	−65.8 (4)	C17—C12—S2—C18	0.9 (2)
C4A—C5—C6—N1	162.8 (4)	C13—C12—S2—C18	−178.4 (2)
N2—C7—N1—C6	178.3 (3)	O4—C26—C25—C24	4.3 (4)
S1—C7—N1—C6	−0.5 (3)	O3—C26—C25—C24	−174.1 (3)
C1—C6—N1—C7	0.6 (3)	C26—C25—C24—C23	170.2 (3)
C5—C6—N1—C7	−179.0 (2)	C25—C24—C23—O2	−18.9 (4)
N2—C7—S1—C1	−178.5 (3)	C25—C24—C23—O1	163.5 (3)

### Bis(2-amino-4,4,7,7-tetramethyl-4,5,6,7-tetrahydro-1,3-benzothiazol-3-ium) succinate–2-amino-4,4,7,7-tetramethyl-4,5,6,7-tetrahydro-1,3-benzothiazol-3-ium hydrogen succinate 4,4,7,7-tetramethyl-3a,5,6,7a-tetrahydrobenzothiazol-2-ylamine (1/1) (V) Hydrogen-bond geometry (Å, º)

**Table:**

*D*—H···*A*	*D*—H	H···*A*	*D*···*A*	*D*—H···*A*
N1—H1*N*···O1	0.93 (3)	1.70 (3)	2.632 (3)	175 (3)
N2—H2*N*···O4^i^	0.89 (4)	1.98 (4)	2.779 (3)	149 (3)
N2—H3*N*···O2	0.82 (4)	1.89 (4)	2.716 (4)	176 (4)
N3—H4*N*···O3	0.87 (8)	1.73 (8)	2.594 (3)	166 (6)
N4—H5*N*···O2^ii^	0.82 (4)	2.07 (4)	2.804 (4)	148 (4)
N4—H6*N*···O4	0.92 (4)	1.93 (4)	2.842 (4)	169 (3)
O3—H1*O*···N3	0.82 (10)	1.78 (10)	2.594 (3)	174 (8)

Symmetry codes: (i) *x*+1, *y*, *z*; (ii) *x*−1, *y*, *z*.
